# Bioinspired balloon catheter integrated with stretchable “flounder” electrodes under high voltage for uniform pulsed field ablation

**DOI:** 10.1126/sciadv.adq5822

**Published:** 2024-12-13

**Authors:** Xuejing Shen, Erwen Jia, Yin Huang, Dingbang Ge, Zheng Sun, Zhiyan Yang, Ping Zhang, Yihao Chen, Xue Feng

**Affiliations:** ^1^ Laboratory of Flexible Electronics Technology, Tsinghua University, Beijing, 100084, China.; ^2^ AML, Department of Engineering Mechanics, Tsinghua University, Beijing, 100084, China.; ^3^ Institute of Flexible Electronics Technology of THU, Zhejiang, Jiaxing, 314000, China.; ^4^ Key Laboratory of Advanced Technologies of Materials, Ministry of Education, School of Materials Science and Engineering, Southwest Jiaotong University, Chengdu, 610031, China.; ^5^ Department of Cardiology, Beijing Tsinghua Changgung Hospital, Beijing, 102218, China.

## Abstract

Atrial fibrillation leads to severe diseases such as heart failure and strokes. While catheter ablation is prevalent for the treatment, existing techniques hardly can achieve both tissue selectivity and ablation uniformity. Here, we propose a bioinspired strategy for balloon-based pulsed field ablation (PFA) systems based on “flounder” electrodes. Inspired by a flounder skeleton and citrus peels, the microfabricated electrodes are ultrathin, stretchable, and have a scattered configuration, withstanding large balloon deformation (87% compression), high voltage (1200 volts), and owning exceptional tissue conformability (720° twists). Mechanical-electrical coupled stimulation optimizes balloon electrodes with hemispherical electric field uniformity. A water lily–inspired transfer printing method enables one-step integration of multielectrodes with the balloon. A comprehensive PFA system is complemented, achieving ablation depths of 3.8 millimeters (potato), 3.1 millimeters (rabbit), and 2.3 millimeters (swine) with good uniformity and electrophysiological isolation. These results shed light on the quantitative design of PFA systems, with high potential for more precise, safe, and effective catheter ablation therapies.

## Teaser

A bionic e-balloon featuring stretchable electrodes is designed for PFA treatment with tissue selectivity and uniform ablation.

## INTRODUCTION

**Fig. 1.**
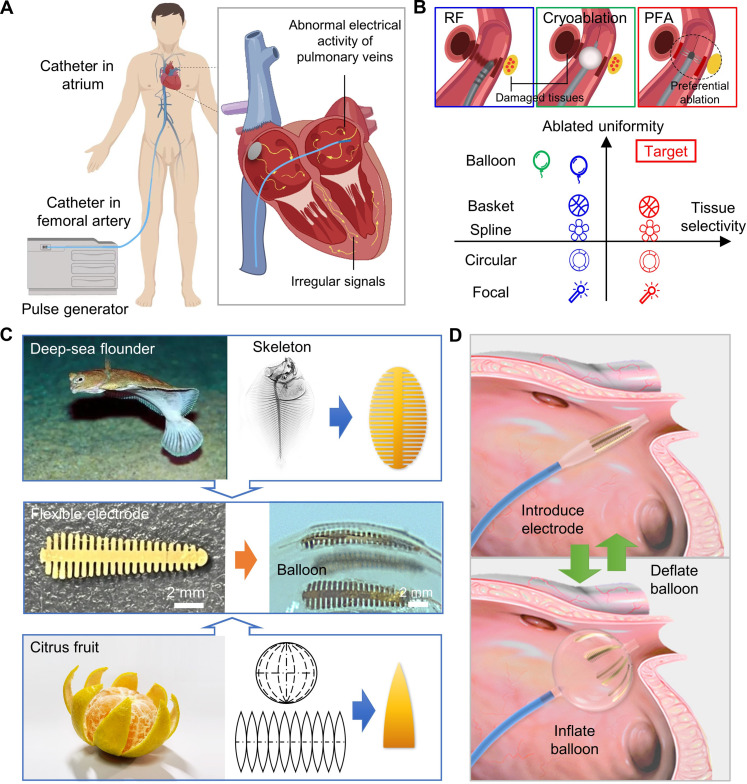
Design concept of balloon electrode of PFA for AF treatment. (**A**) Schematic illustration of catheter ablation for AF treatment. The system consists of an interventional multielectrode catheter and a pulse generator; during the procedure, a catheter is inserted through the femoral artery and advanced into the atrium; electrodes from the catheter target regions with abnormal electrical activity in the pulmonary veins, aiming to restore normal heart rhythm. (**B**) The comparison of ablation principle and distal morphology of RF, cryoablation, and PFA. Thermal ablation of RF (blue box) and cryoablation (green box) damages all tissue types indiscriminately, while PFA was shown to preferential ablation with tissue selectivity. Electrode catheters come in various shapes—focal, circular, spline, basket, and balloon—which influence outcomes by ablated uniformity. (**C**) Design principle of the electrodes inspired by the skeleton of deep-sea flounder and peeling of citrus fruits. (**D**) Schematic illustration of the freely inflated/deflated balloon catheter intervened in the atrium.

Arrhythmias of the heart pose a substantial risk for several cardiovascular complications, including stroke, heart failure, and sudden cardiac death. Among these arrhythmias, atrial fibrillation (AF) is particularly notable for its prevalence and impact on public health (*1*). Until 2019, the worldwide prevalence of AF is approximately 60 million cases and contributes to >8 million disability-adjusted life years (*2*). Catheter ablation applied to pulmonary vein isolation (PVI) is now the most reliable method to treat AF (Fig. 1A) (*3*). Although this is effective, many patients with AF still suffer from a host of adverse effects that include symptomatic AF recurrences (*4*, *5*), collateral injury from thermal energy (*6*, *7*), and cardiac perforation from prolonged procedures (*8*, *9*). Thus, a more efficient and secure method of catheter ablation that is devoid of these adverse effects is desirable.

Typical catheter ablation techniques of AF are categorized on the basis of the energy source used, including radiofrequency (RF) (*10*, *11*), cryoablation (*12*–*15*), and pulsed field ablation (PFA) (*16*, *17*) (Fig. 1B and table S1). While focal RF ablation has been the most commonly used method to achieve PVI to date, its efficiency is limited by the low efficacy of point-by-point ablation, resulting in prolonged duration of the procedure and high requirement for technical experience of physicians (*6*). Contrarily, cryoablation has gained popularity among operators mainly due to its ability to perform PVI in large areas via the cryo-balloon that allows for quick and easy ablation (Fig. 1B, depicted in green) (*12*–*14*). Despite researchers have made ongoing efforts to achieve better efficiency and fewer recurrences via innovating the morphology of electrodes in RF, especially those balloon-based devices (e.g., HotBalloon, Luminize, and Heliostar) (Fig. 1B, depicted in blue) (*18*–*20*), a notable challenge persists. Both techniques of RF and cryotherapy would leave collateral structures at risk for injury due to their thermal basis of energy sources. For instance, phrenic nerve and oesophageal injuries have been reported among 5.0 to 10.0% and 0.1 to 0.5% of patients, respectively (*21*, *22*). The promising solution to those adverse outcomes is the PFA approach, a principle of tissue-selective ablation based on irreversible electroporation (fig. S1) (*16*, *17*). Because of the lower threshold of membrane potential in the myocardium compared to other tissues, PFA can achieve preferential myocardial ablation while sparing sensitive structures, thus improving safety (*16*).

To enhance the ablation efficiency of PFA further, ongoing researches are focused on refining electrode designs, with current iterations including circular [e.g., PulseSelect (*23*–*25*), spline (e.g., FaraPulse) (*26*–*28*), or basket-shaped (e.g., Globe) (*29*–*31*) electrodes; Fig. 1B, depicted in red]. Despite their proven effectiveness, most PFA electrodes presently are rigid, relying on mechanical deformation to transition from a contracted state during catheter insertion to an expanded state within the atrium (*32*). While these electrodes effectively achieve PVI, their stiffness may limit adaptability to varied pulmonary vein anatomies. Furthermore, the uniformity of the electric field is essential for successful AF treatment yet remains inadequately considered and defined. Total conformal tissue contact under complex anatomical structures and even spatial electric field distribution are all crucial for ablated uniformity. Any variability in those aspects could lead to partial ablation, heightening the possibility of AF recurrence. Intuitively, integrated electrodes with an ultrasoft balloon could better adapt to various anatomical curves and may produce more uniform electric fields, thereby facilitating a more effective and safer AF treatment. In 2024, Abbott released the new Volt PFA system with a balloon-in-basket electrode design, where the internal balloon functions as a source of pressure that actively expands the external basket to enhance contact with target tissues. More attempts have been made in the RF systems mentioned above. In particular, the Heliostar from Johnson features patterned multielectrodes exhibiting good conformability between the electrodes and the balloon. Unfortunately, those success stories in RF cannot directly migrate to PFA scenarios, where the design of materials, thickness, geometry, distribution of electrodes, and the current collectors differ significantly from RF ablation to avoid generating uneven electric fields that can cause electrode polarization, electric sparks, and electrical breakdown under high ablation voltages (for comparison of a balloon device for catheter ablation, see table S2) (*33*). To the best of our knowledge, the perfect fusion of PFA electrodes and the soft balloon is still limited. Moreover, none of them own stretchability in the electrodes, which is the key to completely intimate contact with the tissues. Only the perfect fusion of high-voltage, stretchable electrodes with the balloon can result in ideal PFA treatment with ablation uniformity and precise tissue selectivity. The design and manufacture of a fully integrated balloon-based electrode (e-balloon) present difficulties. To recapitulate briefly, the specific challenges in developing the PFA e-balloon are satisfying the electrode’s stretchability and electric field uniformity simultaneously: (i) Despite the basal balloon is stretchable, the integration of hard functional materials, i.e., metal electrodes, hinders its deformability; (ii) these multiple electrodes must be capable of withstanding thousands of volts, as opposed to the hundred volts in RF; their interaction of the electric fields is essential for achieving uniformity and preventing electric sparks; moreover, (iii) the structure, material, and distribution of the electrodes affect both the stretchability and electric field uniformity, where the mechanical and electrical properties of the e-balloon should be coupling designed.

In this study, we propose a bioinspired strategy for material design and heterogeneous integration of balloon-based PFA systems to enable AF treatment with tissue selectivity and ablated uniformity. The designed stretchable high-voltage electrodes exhibit exceptional conformability and deformability. After a mechanical-electrical coupled numerical design, these multiple wired electrodes can be one-step integrated onto the balloon surface by a biomimetic spatial transfer printing method. Our research findings confirm that with an elaborately designed PFA system, the e-balloon–based PFA treatment system is capable of ablating with hemispherical uniformity and achieving effective ablation in the models of vegetables, rabbits, and swine.

## RESULTS

### Bionic inspiration for balloon-based electrodes

Integrating electrodes onto balloons presents substantial challenges due to the contrasting properties of the materials. Electrodes, typically metallic with high modulus and hardness, contrast with the soft, highly contractile balloon materials. This disparity necessitates that an electrode be stretchable and conformable enough to accommodate the large deformations of the balloon during inflating and deflating (*34*). Moreover, the balloon’s three-dimensional (3D) undevelopable surface complicates the integration of two-dimensional (2D) planar electrodes, which must conform intimately to the balloon’s spherical shape without causing wrinkles or stretches.

Drawing inspiration from natural evolution and optimal configurations for enduring complex environments, we designed electrodes modeled after characterizing deep-sea fish bones, i.e., the flounder, as the osteological characteristics of flounders are parallel to the requirement for those metal electrodes; the osteology has evolved to be sufficiently hard to withstand the compressive forces in high-pressure environments while maintaining the necessary flexibility for dynamic adaptation to locomotion. Unlike shallow-water species, deep-sea fish cannot use swim bladders for buoyancy and instead depend on specific lipids. These fish typically have delicate skeletal structures, minimal muscle mass, and high-water content in their bodies, giving their flesh a gelatinous texture. Specifically, the skeleton of the flounder has evolved to be shorter and more scattered, effectively distributing stress and minimizing interface stress between bones and muscles during locomotion, ensuring that the bones remain attached under high pressure and large body deformation (*35*). The contraction process of balloon catheters is similar to the high-water pressure subject to deep-sea flounders, in which scattered, short, and stretchable fishbone-like electrodes make it ideal for withstanding significant balloon deformations while maintaining good attachment between electrodes and target tissues. Thus, we designed the electrode to emulate the skeleton structure of the deep-sea flounder that features a narrow central bone flanked by several slender spines, as shown in Fig. 1C (top). Furthermore, inspiration was drawn from the process of peeling a citrus fruit to achieve better conformal integration of these 2D electrodes with the 3D balloon. As shown in Fig. 1C (bottom), when the peels are evenly removed along the axis of a citrus fruit, the 3D spherical peels can be approximately developed into 2D planar shapes with the top half of each individual unfolded peel resembling a triangle. This spherical unfolding method inspired by citrus peels maximizes the conformance between 2D planes and undevelopable surfaces. Together, by integrating the skeleton of deep-sea flounder with the contour of evenly divided citrus peel, the final shape of the metal electrode was designed as shown in Fig. 1C (middle). After optimizing key parameters of the fishbone-shape electrode, i.e., the height (*h*) and base (*b*) of the triangle, bone width (*w*), and gaps (*g*, width; *n*, numbers), the final structure can adhere perfectly to the balloon surface, deliver a uniform electric field, and ensure safety of the e-balloon (see fig. S2 and table S3 for detailed dimensions). Figure 1D exhibits a final e-balloon with 10 evenly distributed electrodes on its upper hemisphere. This design allows for free expansion and contraction of the balloon. The reason is that the electrodes with low equivalent stiffness do not compromise the balloon’s intrinsic flexibility.

### Mechanical and electrical optimization

**Fig. 2.**
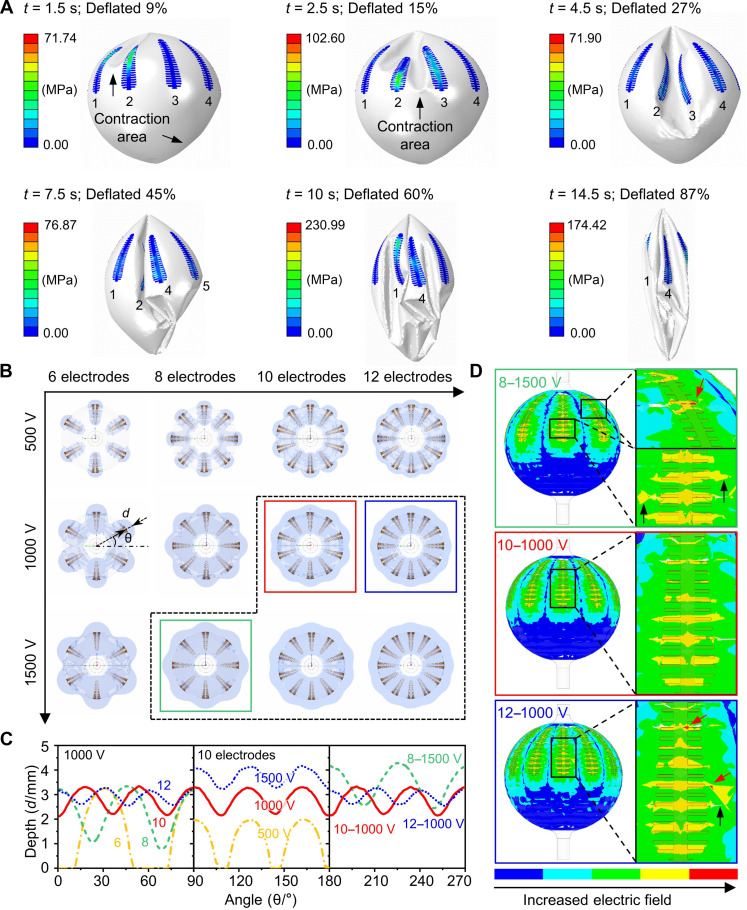
Mechanics and electric field simulations of balloon-based electrodes. (**A**) Simulation results for the deflation process of the balloon electrode showing the distribution of maximum principal tresses at the electrodes. (**B** to **D**) Simulation results for the distribution of electric fields excited by the balloon electrode. (B) The top view of electric field area excited by balloon electrodes with different numbers of electrodes and exciting voltages [the boundary is 400 V/cm; black dashed box indicated configurations with a minimum depth of electric field greater than 2 mm; the different colors of wireframes correspond to the examples in [(C), right] and in (D). (C) The depth of electric field at different circular positions of the balloon with an electric field of 400 V/cm [left: 6 (yellow dashed-dotted line)/8 (green dashed line)/10 (red solid line)/12 (blue dotted line) electrodes with a voltage of 1000 V; middle: 10 electrodes with voltages of 500 V (yellow dashed-dotted line), 1000 V (red solid line), and 1500 V (blue dotted line); right: depths of more than 2 mm (green dashed line: 8 electrodes with voltages of 1500 V labeled “8–1500 V”; red solid line: “10–1000 V”; blue dotted line: “12–1000 V”). (D) The distribution of electric fields on the balloon surface (top: “8–1500 V”; middle: “10–1000 V”; bottom: “12–1000 V”).

The contraction and expansion of a balloon can lead to volume changes exceeding 80%, surpassing the breaking elongation limits of most metals (*36*). This significant deformation poses a high risk of electrode failure. Figure 2A shows a finite element analysis simulation of an e-balloon’s contraction dynamics using a fluid cavity model by controlling balloon volume. The process of a whole contraction lasted about 15 s (movie S1). The balloon is made of thermoplastic polyurethane (TPU; *E* = 100 MPa), while the electrode comprises layers of polyimide (PI), chromium (Cr), and gold (Au; *E* = 40 GPa; play the major role) (see Materials and Methods for details). At 9% deflation, a contraction area appeared on the electrode-less hemisphere, while the upper hemisphere displayed an initial contraction area between two electrodes (labeled as no. 1 and no. 2 in Fig. 2A). Subsequently, at 15% deflation, a new contraction area formed to the right of the no. 2 electrode, causing no. 2 slight bending (maximum stress of 102.6 MPa). When the deflation reached 27%, the no. 2 and no. 3 electrodes rotated inward with less bending, lowering the maximum stress to 71.9 MPa. Until 60%, the maximum stress in electrodes reached 231 MPa, exceeding the tensile strength of Au (127 MPa). Despite this, failure areas in the electrodes were minimal, accounting for less than 1% of the total area and an overall failure rate of only 0.0841%, even when deflated by 87% (see fig. S3 for details). These results suggest that the designed stretchable electrodes can robustly adapt to significant balloon contraction. In addition, the flexibility of the electrodes does not compromise their electrical properties (see fig. S4 for details). These features allow them to maintain reliable functionality even under large mechanical deformation during operations.

Electric field uniformity of the e-balloon should be considered in two directions: circumferential (perpendicular to the catheter) and longitudinal (along the catheter). Figure 2B presents the circumferential electric field distributions for e-balloons with 6, 8, 10, and 12 electrodes at varying voltages (500, 1000, and 1500 V), revealing that uniformity improves as the number of electrodes increases. Analyzing the distance from the 400-V/cm boundary (PFA threshold of the myocardium) (*37*) to the balloon surface indicates that more electrodes result in a flatter depth curve (Fig. 2C, right; from yellow dashed-dotted line to blue dotted line; from 6 to 12 electrodes), where the maximum depth remains stable at around 3.5 mm (with an exciting voltage of 1000 V). Increasing the voltage uniformly shifts the curve upward (Fig. 2C, middle). Hence, targeting depths more than 2 mm, the suitable balloon configurations are outlined with a black dashed line in Fig. 2B; an eight-electrode configuration requires a voltage of 1500 V [green wireframe in Fig. 2B; green dashed line in Fig. 2C (right)], while 10 and 12 electrodes need at least 1000 V [red and blue wireframes in Fig. 2B, respectively; red solid and blue dotted lines in Fig. 2C (right), respectively]. However, higher voltages or too many electrodes can lead to uneven electric fields, potentially causing breakdowns, as indicated in Fig. 2D with local concentrations (yellow areas indicated by black arrows) and extraordinary high points (red areas indicated by red arrows). Therefore, selecting appropriate configurations and voltages is crucial for achieving optimal electric field distribution and ensuring electrode durability. Our preliminary findings endorse an e-balloon with 10 stretchable electrodes as the optimal configuration for achieving uniform electric fields circumferential and longitudinal, covering the entire hemisphere (see fig. S5 and table S4 for more details).

### Rapid fabrication and bionic integration

**Fig. 3.**
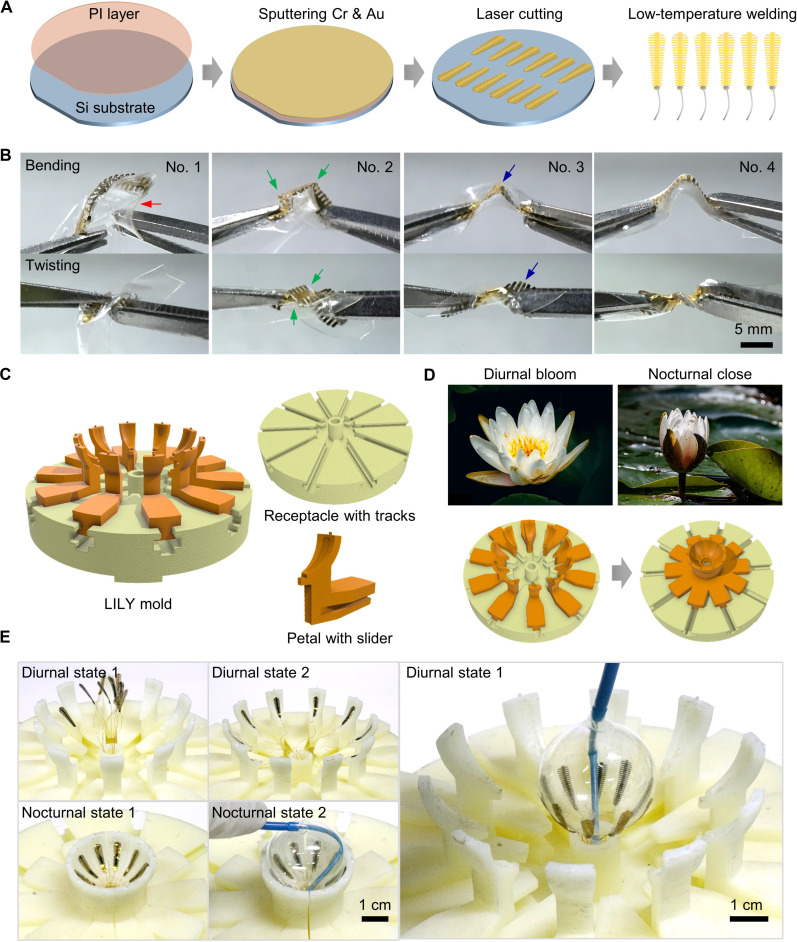
Fabrication and bionic integration process of the e-balloon. (**A**) Fabrication process of the electrodes. (**B**) Bending and twisting tests of electrodes attached to balloon slices with different adhesives (nos.1 to 4). (**C**) Schematic of the LILY mold for the electrode transfer printing. (**D**) Biomimetic principle of LILY mold that mimics the diurnal bloom and nocturnal close of water lilies. (**E**) The process of transfer printing exhibiting two diurnal and two nocturnal states of the LILY mold (from diurnal states 1 and 2 to nocturnal states 1 and 2 and back to diurnal state 1).

The “flounder” electrodes are fabricated in four steps (Fig. 3A): affixing PI films (a thickness of 25 μm) onto silicon wafers; magnetron sputtering sequential layers of 50-nm Cr and 500-nm Au; shaping via laser patterning; low-temperature welding copper (Cu) wires (a diameter of 120 μm) as current collectors. The simplicity of the manufacturing process enables the electrodes for larger-scale production. The flounder electrodes benefit from several optimizations: Magnetron sputtering yields denser, defect-reduced metal clusters with superior metallic phase structures, enabling thinner electrodes that maintain excellent electrical conductivity and adapt to substantial deformations compared to thicker electroplated alternatives (*38*); the high-voltage resistant PI substrate effectively isolates the metal electrodes from the soft balloon, while its “scattered” (patterned) structure with the metal layers ensures electrical isolation without compromising electrode deformability. The scattered defect-free Au layer together with the PI substrate guarantees the electrode’s ability of tolerating high ablation voltage and having stretchability.

Identifying the appropriate adhesive is crucial for establishing a durable and flexible bond between the electrodes and the balloon. The hard-soft heterogeneous interface heightens the risk of debonding and fractures under large deformations. Four adhesives, labeled as no. 1 to no. 4, were evaluated. Equal adhesives (10 μl) were used to bond electrodes and balloon slices, followed by tests for bending, twisting, and peeling (Fig. 3B, figs. S6 to S9, and table S5). There are three typical failures. No. 1 adhesive showed excessive rigidity, limiting deformability and elasticity (indicated by the red arrow; see fig. S6). No. 2 adhesive was brittleness, causing localized folds and stress concentration (green arrows). No. 3 adhesive had poor stickiness, leading to easy debonding (blue arrows). In contrast, the no. 4 adhesive performed optimally, demonstrating smooth deformation and withstanding 720° of twisting (maximum stress of 103 MPa; for mechanical analyses of the electrode deformation, see fig. S8). Increasing its amount further improved interfacial strength without compromising deformability (see fig. S9). The ideal adhesive must not only provide a robust bond at the electrode-balloon heterogeneous interface but also preserve the e-balloon’s stretchability through mechanical matching capabilities.

Transferring multiple electrodes onto the balloon surface poses substantial challenges, including (i) accurate positioning on a spherical surface, (ii) labor-intensive attachment of numerous electrodes, and (iii) achieving conformability with a 3D curvy surface. Conventional planar transfer printing techniques are unsuitable, and current curvy surface transfer methods using stamps are complex and time-consuming (*39*). Inspired by the nyctinasty of the water lily, we developed the LILY (layering, interfacing, latching, and yielding) mold for a one-step spatial integration of multiple electrodes. The LILY mold consists of movable petal-like sliders and a receptacle-like base with tracks (Fig. 3C). Initially, sliders spread outward like a diurnal blooming water lily (Fig. 3D). Electrodes are threaded through the base’s center, resembling pistils (diurnal state 1, Fig. 3E). The bloomed LILY enables placing electrodes into specific grooves on each petal, ensuring precise positioning (diurnal state 2). As the LILY closes, the sliders move inward, aligning the electrodes for transfer onto the balloon (nocturnal state 1). After applying adhesive, the catheter’s head is positioned at the base center, ensuring precise initial placement. The inflated balloon then actively contacts the surrounding electrodes for evenly and simultaneously adhesion under stable pressure (nocturnal state 2). Once set, the mold reopens and leaves the electrodes precisely bonded to the contour of the balloon (LILY reopens to diurnal state 1). This LILY transfer printing strategy facilitates accurate placement of multiple wired electrodes at one time, offering a blueprint for future biomedical applications that require spatial integration of multiple devices onto a 3D curvy surface.

### Free deformability and PFA effectiveness

**Fig. 4.**
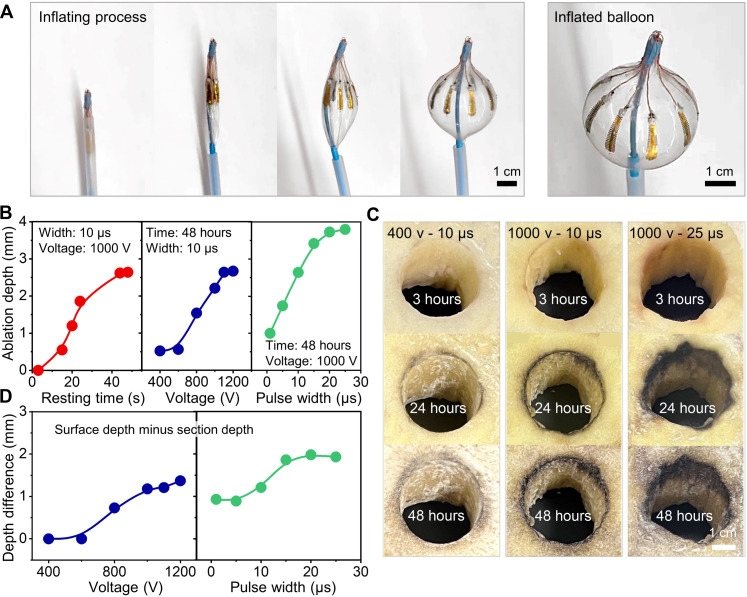
Demonstration of balloon inflation and PFA effect variation on vegetable models. (**A**) Inflating process of a balloon electrode deployed from a mock catheter. (**B** to **D**) PFA effect verification on potatoes. (B) Influencing factors of ablation depth including resting time, voltage, and pulse width. (C) Cross-reference of the voltage, pulse width, and resting time. All potatoes after PFA show circular uniform ablation lesions. (D) The difference of ablation depths between the surface and the section of potatoes after PFA tends to be stable with the increase of voltages and pulse widths.

To construct a comprehensive balloon-based PFA treatment system, we customized an interventional system (including adjustable sheaths and interatrial puncture needle) and a pulse generator (voltage: 0 to 3000 V, pulse width: 1 to 50 μs) (see figs. S10 and S11). The inflation process of an e-balloon is demonstrated in movie S1 and captured in Fig. 4A. The e-balloon can contract and deploy from the catheter with an inner diameter of 4 mm, which is equivalent to a 14F medical catheter sheath. This e-balloon can expand in response to gas/liquid infusion, allowing for adjustable balloon diameters based on requirements, with a diameter of ~28 mm when fully inflated. Although catheter electrodes are typically designed as single-use medical devices, fatigue tests involving more than 10 cycles of inflating, deflating, and movement within the catheter provide evidence of the robustness and reliability of the balloon-based electrodes, guaranteeing stable performance during the procedure.

Before animal testing, the PFA parameters were optimized on vegetable models. Potatoes served as the simulated tissue, each prepared with a hole matching the diameter of the fully inflated balloon and immersed in saline for ablation testing [results in Fig. 4 (B to D), fig. S12, and table S6]. When pulsed electric fields were applied, the electroporation of the cell membranes released a phenoloxidase enzyme that can promote the oxidation of phenolic compounds to yield dark regions (*40*). Then, the dark lesions become visible. The changes were tracked by measuring the depth of these dark regions over time (fig. S12). Figure 4B (left) shows an upward trend of ablation depths over the resting time, where the ablation depth reached 1.86 mm after 24 hours and increased to 2.64 mm after 48 hours (under PFA with a voltage of 1000 V, a frequency of 1 Hz, a width of 10 μs, and a number of 20 times). Measurements beyond 48 hours showed severe dehydration of potatoes, leading to inaccurate results.

In particular, the electroporation, characterized by increased cell membrane permeability in response to electric pulses, is primarily governed by the external electric field that establishes the transmembrane potential across the plasma membrane. Depending on the magnitude of this potential, electroporation can have varying outcomes: It may leave the membrane intact, reversibly permeabilize it (allowing cells to survive), or irreversibly permeabilize it, resulting in cell death (fig. S1) (*16*). This process is analogous to charging a capacitor, where charge accumulation depends on both the strength of the electric field and the duration of exposure. Consequently, the effects of PFA are directly modulated by adjusting voltage and pulse width that emerge as critical parameters influencing the ablation depth. Voltage directly affects the transmembrane potential, while pulse width determines the duration of exposure to the electric field. Both parameters showed a positive, monotonic correlation with ablation depth, as illustrated in Fig. 4B. However, earlier simulations indicated that excessive voltage can lead to localized high electric field intensities (Fig. 2B, highlighted as red regions). Furthermore, excessively long pulse durations increased the risk of thermal damage due to heat accumulation (*16*). To balance ablation efficacy and safety, an optimal voltage range of 800 to 1200 V and pulse widths of 10 to 30 μs were identified. These settings provided effective ablation while mitigating adverse effects such as electrical arcing and heat-related tissue damage (the total energy delivered amounts to 4 J with a power output of 0.2 W). Figure 4C presents a cross-reference of voltage, pulse width, and resting time, demonstrating that ablation depth increases with higher voltage and longer pulse width. Measurements taken at both the surface and 1 cm below the surface revealed that, while higher voltages and pulse widths initially increased the disparity between surface and section depths, this effect plateaued, ensuring uniform ablation across layers.

Observations of uniform dark lesions and consistent ablation depths in the potato model validate the efficacy of the e-balloon in achieving uniform PFA. The optimal parameters—voltages between 800 and 1200 V and pulse widths of 10 to 30 μs—produced ablation depths of at least 2 mm, which are appropriate for effective tissue ablation in clinical applications.

### In vivo validation on animals

**Fig. 5.**
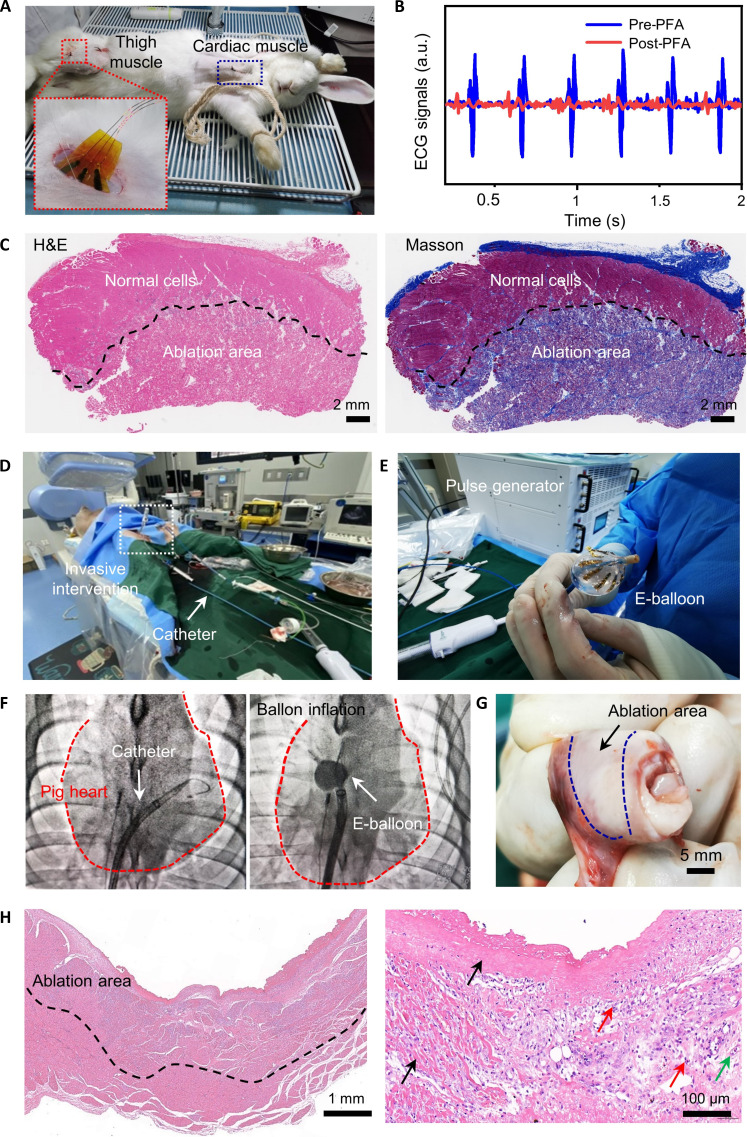
In vivo animal experiments. (**A**) PFA on thigh and cardiac muscles of a rabbit. (**B**) An attenuation of electrocardiogram (ECG) signal from the rabbit after PFA on cardiac muscle. (**C**) Histological sections of thigh muscle after PFA from a rabbit (underwent H&E and Masson’s trichrome stains). The dashed line indicates the ablation lesion boundary. (**D**) The catheter intervened in an adult swine. (**E**) The self-developed pulse power and a balloon electrode that has completed interventional PFA. (**F**) Intraoperative real-time imaging with catheter intervention and balloon inflation in the right superior pulmonary vein of a swine. (**G**) Everted appendage of the pulmonary vein after PFA from a swine. (**H**) Histological sections of the pulmonary vein after PFA from a swine. The dashed line shows the ablation lesion boundary. a.u., arbitrary units.

In vivo experiments were carried out in rabbits and a swine, the former performing extracardiac myocardial ablation by thoracotomy, and the latter performing intracardiac myocardial ablation by an interventional method. New Zealand White rabbits (weighed ~2.5 kg) underwent PFA using a planar electrode matching the e-balloon dimensions (Fig. 5A, insert). Postanesthesia, thoracotomy was performed, and the extracardiac myocardium was exposed for ablation operations (for the surgery scene, see fig. S13). Real-time electrocardiogram (ECG) monitoring from both sides of the ablation area during surgery confirmed the effective blockade and isolation of cardiac electrical signals (Fig. 5B). Additional PFA tests were conducted on various locations of leg muscles (for electrical pulses acting process, see movie S4). After a 3-day survival period, the rabbits were euthanized, and the ablated tissues were processed for histological analysis (Fig. 5C and fig. S14). Hematoxylin and eosin (H&E) staining showed normal tissue in the pale red and ablated area with disrupted cell membranes in irregular aggregations (Fig. 5C, left). Masson’s trichrome staining highlighted scar tissue in blue, demonstrating clear ablation boundaries and uniform areas (Fig. 5C, right). PFA efficacy was evidenced by an ablation depth of 2.77 mm (using a voltage of 800 V, a pulse width of 10 μs, and a pulse number of 50). More ablation depths corresponding to pulse parameters were detailed in table S7, with the maximum depth recorded at 3.1 mm.

The interventional treatment capability and effectiveness of the balloon-based PFA system were validated using a 51-kg swine (Fig. 5D). The electrode catheter was inserted through the hind leg of the swine and connected to the external pulse generator (Fig. 5E and fig. S15A). It traversed the femoral vein, inferior vena cava, and interatrial septum, reaching the left atrium and the right superior pulmonary vein. The placement and progression of the catheter were monitored by real-time computed tomography fluoroscopy (Fig. 5F). Then, the e-balloon was intervened through the catheter, inflated to a diameter of 28 mm, and delivered PFA (voltage: 1000 V, pulse width: 10 μs) without perforation, bleeding, or any abnormalities during the whole operations. The postprocedure swine was monitored for 3 days before euthanasia. Examination of the pulmonary vein revealed effective PFA and uniform ablation as evidenced by the whitening of everted appendage at ablated sites (Fig. 5G). The histological assessment confirmed a clear ablation boundary at a depth of 1.81 mm (Fig. 5H). Extensive necrosis within the medial and adventitial layers of the blood vessel resulted in the blurring of the structural boundaries between these layers, where the nucleus underwent fragmentation and dissolution, appearing as homogeneous substances (marked by black arrows). In addition, the adventitial layers showed modest proliferation of fibroblasts and sparse infiltration of lymphocytes and granulocytes (red arrows), with a few neoangiogenic vessels visible (indicated by the green arrow). Together, the balloon-based PFA system has been demonstrated to be an effective ablation technique. Further ablations in the atrium dextrum confirmed that the system can achieve depths of up to 2.3 mm in swine (fig. S15 and table S8). Moreover, the capacity of circumferential uniform ablation of the e-balloon was substantiated by the ablation boundary that illustrated consistent uniformity across an 8-mm span, exceeding the separation distance between two electrodes in the balloon (Fig. 5H and fig. S16).

## DISCUSSION

This study innovates a strategy for the achievement of both tissue selectivity and hemispherical uniformity for PFA. A system-level bionic design approach is proposed for the construction of the PFA balloon-based electrode, emphasizing the integration of stretchability and electric field uniformity. From a structure standpoint, the stretchable electrodes, inspired by the flounder skeleton and citrus peels, offer exceptional conformability and deformability; a biomimetic transfer printing method (LILY mold, inspired by the water lily) enables rapid and accurate integration of multiple wired electrodes onto the balloon surface; a mechanical-electrical numerical design strategy for balloon-shaped electrodes addresses large deformations, achieving a low failure rate of 0.0841% under 89% balloon compression, and ensures uniform ablation with hemispherical electric field uniformity at high voltages. From a material perspective, we propose a scalable manufacturing solution that integrates stretchable electrodes with the soft balloon, addressing mechanical-electrical matching challenges, which involves using magnetron sputtering to prepare thinner and high-quality electrodes, applying a high dielectric strength substrate to isolate high voltages while maintaining stretchability, and identifying suitable interfacial medium to achieve optimal bonding strength and tress matching. The efficacy of the PFA system was validated in potato experiments, demonstrating consistent circular ablation with a depth of up to 3.8 mm under 1000 V and 25 μs pulses. Rabbit experiments confirmed effective electrophysiological isolation and an ablation depth of up to 3.1 mm with a single shot. Swine experiments with the interventional PFA system further validated the balloon electrode’s uniform ablation capability, achieving uniformity across an 8-mm span (exceeding the electrode separation within the balloon) and an ablation depth of up to 2.3 mm. A comparison highlighting our device against the morphology, characteristics, and animal experimental results of other clinical PFA catheters is provided in table S9. The balloon’s inherent conformability can ensure better contact with the pulmonary vein ostia and atrial walls, allowing the system to deliver uniform and effective ablation without multiple reorientations, thus resulting in substantial time savings and improved procedure efficiency. This study, from standpoints of both the structure and the material, not only offers a promising catheter ablation method with superior performance but also advances the understanding of stretchable balloon electrodes for the design and development of future 3D electronics in biomedical instruments.

### Outlook

The current system demonstrates an effective approach for selective PFA, providing a flexible platform adaptable to other clinical applications. Current clinical practice often uses stand-alone mapping systems, which provide precise, real-time electroanatomic data independently of the ablation catheter. Systems such as CARTO (Biosense Webster) and EnSite (Abbott) are widely used alongside various ablation technologies, including PFA, due to their high-resolution mapping capabilities and compatibility with multiple catheter types. Our PFA system after integrated these dedicated mapping systems might offer detailed localization and tracking before and during ablation, optimizing accuracy and flexibility across different procedural stages. Besides, integrating multimodal sensing technologies (e.g., electrophysiological mapping) would enable simultaneous cardiac signal monitoring during ablation, offering real-time heart rhythm data and facilitating personalized treatments (*41*–*43*). The stretchable balloon electrodes can enhance tissue contact, thus improving the accuracy of signal recordings. In addition, recording signal changes within multiple electrodes could possibly sense tissue contact pressure. Coupled with temperature sensors, these features would provide real-time temperature feedback on tissue interactions, enhancing safety by preventing over-ablation and optimizing treatment outcomes. This work lays the foundation for balloon-based PFA devices with flexible electrodes, potentially enhancing AF treatment efficacy and patient quality of life. The principles outlined here have broader implications, including conformal electrode design, multimodal integration, 3D electronic fabrication, and adaptable designs for various surgical procedures using inflatable balloon catheters.

### Limitations

Several limitations must be addressed to establish the clinical viability of the balloon-based PFA system. Now, the device remains in the feasibility stage, with limited in vivo studies. Broader validation on more animal models like AF swine models is necessary. The ultimate translation of this technique into a viable clinical product depends on rigorous human clinical trials. Moreover, the durability of the system during extended use, particularly concerning lesion continuity and gap prevention during procedures like PVI, also requires additional evaluation. Further optimization of electrode design, along with the integration of advanced technologies (e.g., 3D electroanatomic mapping and real-time visualization) will be crucial for enhancing the tissue-electrode contact and impedance readings, thereby facilitating more reliable lesion formation and precise ablation procedures. Besides, safe energy delivery parameters for the PFA of myocardium might be evaluated in more detail. These advancements would be essential for moving the system toward clinical application and improving patient outcomes.

## MATERIALS AND METHODS

### Fabrication of the balloon-based PFA system

#### Stretchable electrodes

The high-voltage resistant PI film (a thickness of 25 μm) was chosen as the isolation between the balloon and the electrodes, which is attached to a silicon wafer. Next, using magnetron sputtering, a 50-nm layer of Cr and then a 500-nm layer of gold (Au) are applied on top of the PI film, where the thin Cr layer was used to increase the bond between Au and the PI substrate. After all layers were deposited, the film was cut into designed shapes of electrodes using laser cutters. The laser patterning of the PI substrate with the metal ensured electrical isolation without affecting the deformability of the electrodes. The final step involves attaching a copper (Cu) wire (a diameter of 120 μm) to the electrodes by low-temperature welding serving as the current collector.

#### Balloon-based PFA system

A custom LILY transfer mold was used to transfer the multiple electrodes to the TPU balloon surface, enabling the simultaneous application of all electrodes in a single step. The TPU balloons used in this study were procured from Kossel Medtech Co. Ltd. (Suzhou, China). An appropriate amount of adhesive (S8005N, Alders, Germany) was used to bond the electrodes and the balloon. The design of the catheter’s handle incorporated features for balloon inflation and unidirectional deflection, facilitating optimal delivery and accurate positioning at the pulmonary vein. To support the application of microsecond-level high-voltage pulses, a specialized power generator was developed. This device is capable of adjusting critical parameters such as voltage (0 to 3000 V), pulse width (1 to 50 μs), and pulse count, featuring single-channel, time-divided asynchronous discharge capabilities. It integrates display and control functions and offers interoperability with other systems, including ECG modules, enhancing its utility and adaptability in experimental setups.

### Computational methods

#### Mechanical simulation

The commercial software ABAQUS is used for finite element analysis of the mechanical deformations of the balloon contractions. The simulations were carried out using explicit dynamics to compute the 3D shell model, ensuring solution accuracy through five integration points across the thickness of each material layer. The balloon’s specifications included a diameter of 28 mm, a thickness of 20 μm, an elastic modulus of 100 MPa, a Poisson’s ratio of 0.45, and a density of 1500 kg/m^3^. The electrode model comprised materials from the inside out as follows: PI with a thickness of 25 μm, an elastic modulus of 2.5 GPa, a Poisson’s ratio of 0.34, and a density of 1420 kg/m^3^; Cr with a thickness of 50 nm, an elastic modulus of 140 GPa, a Poisson’s ratio of 0.21, and a density of 7190 kg/m^3^; and gold (Au) with a thickness of 500 nm, an elastic modulus of 40 GPa, a Poisson’s ratio of 0.44, and a density of 19,280 kg/m^3^. A fluid cavity was used to simulate the deflation process of the balloon, controlling the balloon’s volume. One end of the balloon is fixed, while the other end can only move along the center axis. The initial volume of the balloon was set at 11,493.7 mm^3^, reducing to 1235.7 mm^3^ at 14.86 s, with the end of the balloon extended by 18 mm along the center axis. The simulation settings included general contact, with normal hard contact and tangential frictionless conditions.

#### Electric field simulations

The commercial software Maxwell 3D is used for electric field simulations. The geometric parameters of the balloon and electrodes were defined according to actual dimensions, with the balloon’s diameter established at 28 mm, and electrode specifics were provided in table S3. To explore the influence of electrode quantity on the electric field distribution, configurations featuring 6, 8, 10, and 12 electrodes, equally spaced along the balloon’s perimeter, were evaluated. The investigation encompassed a range of applied voltages, specifically 500, 1000, and 1500 V, to discern the impact of varying electrical potentials. The simulations were contextualized within an environment mimicking blood. The TPU composing the balloon was assigned a resistivity of 6 × 10^14^ ohm·cm, whereas the electrodes were characterized by a resistivity of 12.9 × 10^−6^ ohm·cm and a relative permittivity of 12.

### Characterization of balloon electrodes

#### Bending, twisting, and peeling test

The no. 1 adhesive (LOCTITE AA 3311) was purchased from Henkel (Germany). The no. 2 adhesive (Ergo 5210) was purchased from Ergo (Switzerland). The no. 3 adhesive was purchased from Compont (China). The no. 4 adhesive (S8005N) was purchased from Alders (Germany). The TPU was cut to required dimensions followed by the application of adhesives for bonding the electrodes to the TPU surface. In bending and twisting tests, the amount of adhesive used was 10 μl in all cases. The peeling strength between the electrodes and balloon surface was evaluated according to the Chinese standard (GB 18173.1-2012) with a peel speed of 50 mm/min.

#### Potato experiments

A cylindrical hole with a diameter of 28 mm was created in each potato to establish a standardized area for ablation trials. Following preparation, the potatoes were immersed in a physiological saline solution, creating an environment akin to the conductive properties of biological tissues undergoing PFA. Ablation trials were conducted across a voltage range of 400 to 1200 V. Pulse widths were varied between 1 and 25 μs to examine the influence of pulse duration on the efficacy of ablation. A total of 12 potatoes were tested, and for each potato, ablation depth was measured at five different circumferential locations. The average of these measurements was taken as the representative depth for each sample. The depth of ablation was measured at subsequent intervals following the procedure: 3, 15, 24, and 48 hours.

### Animal experiments

#### Rabbit experiments

The tests were performed at Hangzhou HuaZhu Biotechnology (Hangzhou, China) and approved by the Ethics Committee (no. ZJCLA-IACUC-20050071). Experiments selected New Zealand White rabbits, weighing 2.5 kg. Rabbits underwent muscle injection anesthesia, were immobilized, shaved, disinfected with iodophor, and received local anesthesia at the thoracic area before thoracotomy and electrocautery for hemostasis. After exposing the thoracic cavity and pericardium, physiological saline was injected at the surgical site. The treatment electrode was placed against the heart/thigh surface for ablation. Posttreatment, the chest was sutured closed and disinfected. Rabbits were observed for 3 days before tissue collection for H&E and Masson’s trichrome staining.

#### Swine experiments

The tests were performed at Gateway Medical Innovation Center (Shanghai, China) and approved by the Ethics Committee (no. SH2022-12003). Adult swine weighing 51 kg underwent a minimum of 3-day quarantine and were fasted for 12 hours before surgery to empty the stomach contents, although water was not withheld. A 14F adjustable sheath (Fustar, Guangdong, China) was used to perform femoral vein puncture for right atrial access, followed by transseptal puncture (407200, Abbot, Shanghai, China) to enter the left atrium. The balloon catheter was inserted into the sheath and was inflated with 10 ml of a saline and contrast mixture to a diameter of 28 mm. The electrode connected a high-voltage pulse generator, which delivered ablation pulses as per predefined parameters. Animals were euthanized 3 days postprocedure for histopathological examination.

#### Histopathological examination

Specimens for histopathological examination were fixed in 4% paraformaldehyde and processed according to the standard histopathological procedures including trimming, dehydration, embedding, sectioning, staining, and coverslipping. Whole slide imaging was performed using a Pannoramic scanner (3DHISTECH, Hungary) and viewed with CaseViewer 2.4 software. Image analysis was conducted using Image-Pro Plus 6.0 (Media Cybernetics, USA).

## Supplementary Materials

### The PDF file includes:

Figs. S1 to S16 Tables S1 to S9 Legends for movies S1 to S4 References

## References

[1] G. Y. H. Lip, H. F. Tse, D. A. Lane, Atrial fibrillation. Lancet 379, 648–661 (2012). 22166900 10.1016/S0140-6736(11)61514-6

[2] A. D. Elliott, M. E. Middeldorp, I. C. Van Gelder, C. M. Albert, P. Sanders, Epidemiology and modifiable risk factors for atrial fibrillation. Nat. Rev. Cardiol. 20, 404–417 (2023). 36600003 10.1038/s41569-022-00820-8

[3] H. Calkins, G. Hindricks, R. Cappato, Y.-H. Kim, E. B. Saad, L. Aguinaga, J. G. Akar, V. Badhwar, J. Brugada, J. Camm, P.-S. Chen, S.-A. Chen, M. K. Chung, J. Cosedis Nielsen, A. B. Curtis, D. W. Davies, J. D. Day, A. d’Avila, N. M. S. de Groot, L. Di Biase, M. Duytschaever, J. R. Edgerton, K. A. Ellenbogen, P. T. Ellinor, S. Ernst, G. Fenelon, E. P. Gerstenfeld, D. E. Haines, M. Haissaguerre, R. H. Helm, E. Hylek, W. M. Jackman, J. Jalife, J. M. Kalman, J. Kautzner, H. Kottkamp, K. H. Kuck, K. Kumagai, R. Lee, T. Lewalter, B. D. Lindsay, L. Macle, M. Mansour, F. E. Marchlinski, G. F. Michaud, H. Nakagawa, A. Natale, S. Nattel, K. Okumura, D. Packer, E. Pokushalov, M. R. Reynolds, P. Sanders, M. Scanavacca, R. Schilling, C. Tondo, H.-M. Tsao, A. Verma, D. J. Wilber, T. Yamane, C. Blomström-Lundqvist, A. A. V. De Paola, P. M. Kistler, G. Y. H. Lip, N. S. Peters, C. F. Pisani, A. Raviele, E. B. Saad, K. Satomi, M. K. Stiles, S. Willems, 2017 HRS/EHRA/ECAS/APHRS/SOLAECE expert consensus statement on catheter and surgical ablation of atrial fibrillation. Europace 20, e1–e160 (2018). 10.1093/europace/eux274 PMC5834122 29016840

[4] D. S. Chew, E. Black-Maier, Z. Loring, P. A. Noseworthy, D. L. Packer, D. V. Exner, D. B. Mark, J. P. Piccini, Diagnosis-to-ablation time and recurrence of atrial fibrillation following catheter ablation. Circ. Arrhythm. Electrophysiol. 13, e008128 (2020). 32191539 10.1161/CIRCEP.119.008128 PMC7359927

[5] J. L. Pallisgaard, G. H. Gislason, J. Hansen, A. Johannessen, C. Torp-Pedersen, P. V. Rasmussen, M. L. Hansen, Temporal trends in atrial fibrillation recurrence rates after ablation between 2005 and 2014: A nationwide Danish cohort study. Eur. Heart J. 39, 442–449 (2018). 29020388 10.1093/eurheartj/ehx466

[6] D. Spragg, Collateral damage during ablation of atrial fibrillation—Lessons learnt in the past decade. J. Atr. Fibrillation 4, 478 (2012). 28496719 10.4022/jafib.478 PMC5153008

[7] M. El Baba, D. Sabayon, M. Refaat, Radiofrequency catheter ablation: How to manage and prevent collateral damage? J. Innov. Card. Rhythm Manag. 11, 4234–4240 (2020). 32983592 10.19102/icrm.2020.110901 PMC7510472

[8] H.-S. Mun, B. Joung, J. Shim, H. J. Hwang, J. Y. Kim, M.-H. Lee, H.-N. Pak, Does additional linear ablation after circumferential pulmonary vein isolation improve clinical outcome in patients with paroxysmal atrial fibrillation? Prospective randomised study. Heart 98, 480–484 (2012). 22285969 10.1136/heartjnl-2011-301107 PMC3285139

[9] A. Verma, C. Jiang, T. R. Betts, J. Chen, I. Deisenhofer, R. Mantovan, L. Macle, C. A. Morillo, W. Haverkamp, R. Weerasooriya, J.-P. Albenque, S. Nardi, E. Menardi, P. Novak, P. Sanders, STAR AF II Investigators, Approaches to catheter ablation for persistent atrial fibrillation. N. Engl. J. Med. 372, 1812–1822 (2015). 25946280 10.1056/NEJMoa1408288

[10] M. R. Williams, J. R. Stewart, S. F. Bolling, S. Freeman, J. T. Anderson, M. Argenziano, C. R. Smith, M. C. Oz, Surgical treatment of atrial fibrillation using radiofrequency energy. Ann. Thorac. Surg. 71, 1939–1944 (2001). 11428388 10.1016/s0003-4975(01)02594-2

[11] A. Njoku, M. Kannabhiran, R. Arora, P. Reddy, R. Gopinathannair, D. Lakkireddy, P. Dominic, Left atrial volume predicts atrial fibrillation recurrence after radiofrequency ablation: A meta-analysis. Europace 20, 33–42 (2018). 28444307 10.1093/europace/eux013

[12] K.-H. Kuck, J. Brugada, A. Fürnkranz, A. Metzner, F. Ouyang, K. R. J. Chun, A. Elvan, T. Arentz, K. Bestehorn, S. J. Pocock, J.-P. Albenque, C. Tondo, for the FIRE AND ICE Investigators, Cryoballoon or radiofrequency ablation for paroxysmal atrial fibrillation. N. Engl. J. Med. 374, 2235–2245 (2016). 27042964 10.1056/NEJMoa1602014

[13] J. G. Andrade, P. Khairy, P. G. Guerra, M. W. Deyell, L. Rivard, L. Macle, B. Thibault, M. Talajic, D. Roy, M. Dubuc, Efficacy and safety of cryoballoon ablation for atrial fibrillation: A systematic review of published studies. Heart Rhythm 8, 1444–1451 (2011). 21457789 10.1016/j.hrthm.2011.03.050

[14] J. G. Andrade, Cryoballoon ablation for pulmonary vein isolation. J. Cardiovasc. Electrophysiol. 31, 2128–2135 (2020). 32239557 10.1111/jce.14459

[15] J. P. Erinjeri, T. W. I. Clark, Cryoablation: Mechanism of action and devices. J. Vasc. Interv. Radiol. 21, S187–S191 (2010). 20656228 10.1016/j.jvir.2009.12.403 PMC6661161

[16] A. Verma, S. J. Asivatham, T. Deneke, Q. Castellvi, R. E. Neal II, Primer on pulsed electrical field ablation: Understanding the benefits and limitations. Circ. Arrhythm. Electrophysiol. 14, e010086 (2021). 34538095 10.1161/CIRCEP.121.010086

[17] V. Y. Reddy, P. Neuzil, J. S. Koruth, J. Petru, M. Funosako, H. Cochet, L. Sediva, M. Chovanec, S. R. Dukkipati, P. Jais, Pulsed field ablation for pulmonary vein isolation in atrial fibrillation. J. Am. Coll. Cardiol. 74, 315–326 (2019). 31085321 10.1016/j.jacc.2019.04.021

[18] C. Gianni, Q. Chen, D. Della Rocca, U. Canpolat, H. Ayhan, B. MacDonald, S. Mohanty, C. Trivedi, A. Natale, A. Al-Ahmad, Radiofrequency balloon devices for atrial fibrillation ablation. Card. Electrophysiol. Clin. 11, 487–493 (2019). 31400873 10.1016/j.ccep.2019.05.009

[19] D.-H. Kim, N. Lu, R. Ghaffari, Y.-S. Kim, S. P. Lee, L. Xu, J. Wu, R.-H. Kim, J. Song, Z. Liu, J. Viventi, B. de Graff, B. Elolampi, M. Mansour, M. J. Slepian, S. Hwang, J. D. Moss, S.-M. Won, Y. Huang, B. Litt, J. A. Rogers, Materials for multifunctional balloon catheters with capabilities in cardiac electrophysiological mapping and ablation therapy. Nat. Mater. 10, 316–323 (2011). 21378969 10.1038/nmat2971 PMC3132573

[20] M. Han, L. Chen, K. Aras, C. Liang, X. Chen, H. Zhao, K. Li, N. R. Faye, B. Sun, J. H. Kim, W. Bai, Q. Yang, Y. Ma, W. Lu, E. Song, J. M. Baek, Y. Lee, C. Liu, J. B. Model, G. Yang, R. Ghaffari, Y. Huang, I. R. Efimov, J. A. Rogers, Catheter-integrated soft multilayer electronic arrays for multiplexed sensing and actuation during cardiac surgery. Nat. Biomed. Eng. 4, 997–1009 (2020). 32895515 10.1038/s41551-020-00604-w PMC8021456

[21] A. Deshmukh, N. J. Patel, S. Pant, N. Shah, A. Chothani, K. Mehta, P. Grover, V. Singh, S. Vallurupalli, G. T. Savani, A. Badheka, T. Tuliani, K. Dabhadkar, G. Dibu, Y. M. Reddy, A. Sewani, M. Kowalski, R. Mitrani, H. Paydak, J. F. Viles-Gonzalez, In-hospital complications associated with catheter ablation of atrial fibrillation in the United States between 2000 and 2010. Circulation 128, 2104–2112 (2013). 24061087 10.1161/CIRCULATIONAHA.113.003862

[22] M. Mansour, D. Lakkireddy, D. Packer, J. D. Day, S. Mahapatra, K. Brunner, V. Reddy, A. Natale, Safety of catheter ablation of atrial fibrillation using fiber optic–based contact force sensing. Heart Rhythm 14, 1631–1636 (2017). 28734985 10.1016/j.hrthm.2017.07.023

[23] A. Verma, L. Boersma, D. E. Haines, A. Natale, F. E. Marchlinski, P. Sanders, H. Calkins, D. L. Packer, J. Hummel, B. Onal, S. Rosen, K.-H. Kuck, G. Hindricks, B. Wilsmore, First-in-human experience and acute procedural outcomes using a novel pulsed field ablation system: The PULSED AF Pilot Trial. Circ. Arrhythm. Electrophysiol. 15, e010168 (2022). 34964367 10.1161/CIRCEP.121.010168 PMC8772438

[24] F. H. M. Wittkampf, R. van Es, K. Neven, Electroporation and its relevance for cardiac catheter ablation. JACC Clin. Electrophysiol. 4, 977–986 (2018). 30139498 10.1016/j.jacep.2018.06.005

[25] M. T. Stewart, D. E. Haines, A. Verma, N. Kirchhof, N. Barka, E. Grassl, B. Howard, Intracardiac pulsed field ablation: Proof of feasibility in a chronic porcine model. Heart Rhythm 16, 754–764 (2019). 30385383 10.1016/j.hrthm.2018.10.030

[26] V. Y. Reddy, J. Koruth, P. Jais, J. Petru, F. Timko, I. Skalsky, R. Hebeler, L. Labrousse, L. Barandon, S. Kralovec, M. Funosako, B. B. Mannuva, L. Sediva, P. Neuzil, Ablation of atrial fibrillation with pulsed electric fields: An ultra-rapid, tissue-selective modality for cardiac ablation. JACC Clin. Electrophysiol. 4, 987–995 (2018). 30139499 10.1016/j.jacep.2018.04.005

[27] J. Koruth, K. Kuroki, J. Iwasawa, Y. Enomoto, R. Viswanathan, R. Brose, E. D. Buck, M. Speltz, S. R. Dukkipati, V. Y. Reddy, Preclinical evaluation of pulsed field ablation: Electrophysiological and histological assessment of thoracic vein isolation. Circ. Arrhythm. Electrophysiol. 12, e007781 (2019). 31826647 10.1161/CIRCEP.119.007781 PMC6924932

[28] J. S. Koruth, K. Kuroki, J. Iwasawa, R. Viswanathan, R. Brose, E. D. Buck, E. Donskoy, S. R. Dukkipati, V. Y. Reddy, Endocardial ventricular pulsed field ablation: A proof-of-concept preclinical evaluation. Europace 22, 434–439 (2020). 31876913 10.1093/europace/euz341 PMC7058968

[29] V. Y. Reddy, E. Anter, G. Rackauskas, P. Peichl, J. S. Koruth, J. Petru, M. Funasako, K. Minami, A. Natale, P. Jais, H. Nakagawa, G. Marinskis, A. Aidietis, J. Kautzner, P. Neuzil, Lattice-tip focal ablation catheter that toggles between radiofrequency and pulsed field energy to treat atrial fibrillation. Circ. Arrhythm. Electrophysiol. 13, e008718 (2020). 32383391 10.1161/CIRCEP.120.008718

[30] H. Kottkamp, F. Moser, A. Rieger, D. Schreiber, C. Pönisch, M. Trofin, Global multielectrode contact mapping plus ablation with a single catheter: Preclinical and preliminary experience in humans with atrial fibrillation. J. Cardiovasc. Electrophysiol. 28, 1247–1256 (2017). 28800169 10.1111/jce.13310

[31] J. Koruth, A. Verma, I. Kawamura, D. Reinders, J. G. Andrade, M. W. Deyell, N. Mehta, V. Y. Reddy, PV isolation using a spherical array PFA catheter: Preclinical assessment and comparison to radiofrequency ablation. JACC Clin. Electrophysiol. 9, 652–666 (2023). 36842871 10.1016/j.jacep.2023.01.022

[32] L. Lu, S. Leanza, R. R. Zhao, Origami with rotational symmetry: A review on their mechanics and design. Appl. Mech. Rev. 75, 050801 (2023).

[33] S. P. M. Bane, J. L. Ziegler, J. E. Shepherd, Investigation of the effect of electrode geometry on spark ignition. Combust. Flame 162, 462–469 (2015).

[34] Y. Su, Z. Liu, S. Wang, R. Ghaffari, D.-H. Kim, K.-C. Hwang, J. A. Rogers, Y. Huang, Mechanics of stretchable electronics on balloon catheter under extreme deformation. Int. J. Solids Struct. 51, 1555–1561 (2014).

[35] C. R. Black, P. B. Berendzen, Shared ecological traits influence shape of the skeleton in flatfishes (Pleuronectiformes). PeerJ 8, e8919 (2020). 32280569 10.7717/peerj.8919 PMC7134016

[36] G. E. Dieter, D. Bacon, *Mechanical Metallurgy* (McGraw-hill New York, 1976), vol. 3.

[37] I. Kaminska, M. Kotulska, A. Stecka, J. Saczko, M. Drag-Zalesinska, T. Wysocka, A. Choromanska, N. Skolucka, R. Nowicki, J. Marczak, J. Kulbacka, Electroporation-induced changes in normal immature rat myoblasts (H9C2). Gen. Physiol. Biophys. 31, 19–25 (2012). 22447827 10.4149/gpb_2012_003

[38] P. J. Kelly, R. D. Arnell, Magnetron sputtering: A review of recent developments and applications. Vacuum 56, 159–172 (2000).

[39] X. Chen, W. Jian, Z. Wang, J. Ai, Y. Kang, P. Sun, Z. Wang, Y. Ma, H. Wang, Y. Chen, X. Feng, Wrap-like transfer printing for three-dimensional curvy electronics. Sci. Adv. 9, eadi0357 (2023). 37494444 10.1126/sciadv.adi0357 PMC10371014

[40] M. Hjouj, B. Rubinsky, Magnetic resonance imaging characteristics of nonthermal irreversible electroporation in vegetable tissue. J. Membr. Biol. 236, 137–146 (2010). 20631997 10.1007/s00232-010-9281-2

[41] W. Lee, S. Kobayashi, M. Nagase, Y. Jimbo, I. Saito, Y. Inoue, T. Yambe, M. Sekino, G. G. Malliaras, T. Yokota, M. Tanaka, T. Someya, Nonthrombogenic, stretchable, active multielectrode array for electroanatomical mapping. Sci. Adv. 4, eaau2426 (2018). 30345362 10.1126/sciadv.aau2426 PMC6195340

[42] H. Hu, Y. Ma, X. Gao, D. Song, M. Li, H. Huang, X. Qian, R. Wu, K. Shi, H. Ding, M. Lin, X. Chen, W. Zhao, B. Qi, S. Zhou, R. Chen, Y. Gu, Y. Chen, Y. Lei, C. Wang, C. Wang, Y. Tong, H. Cui, A. Abdal, Y. Zhu, X. Tian, Z. Chen, C. Lu, X. Yang, J. Mu, Z. Lou, M. Eghtedari, Q. Zhou, A. Oberai, S. Xu, Stretchable ultrasonic arrays for the three-dimensional mapping of the modulus of deep tissue. Nat. Biomed. Eng. 7, 1321–1334 (2023). 37127710 10.1038/s41551-023-01038-w

[43] S. Chen, J. Qi, S. Fan, Z. Qiao, J. C. Yeo, C. T. Lim, Flexible wearable sensors for cardiovascular health monitoring. Adv. Healthc. Mater. 10, e2100116 (2021). 33960133 10.1002/adhm.202100116

[44] R. V. Davalos, I. L. M. Mir, B. Rubinsky, Tissue ablation with irreversible electroporation. Ann. Biomed. Eng. 33, 223–231 (2005). 15771276 10.1007/s10439-005-8981-8

[45] F. Straube, U. Dorwarth, J. Pongratz, B. Bruck, M. Wankerl, S. Hartl, E. Hoffmann, The fourth cryoballoon generation with a shorter tip to facilitate real-time pulmonary vein potential recording: Feasibility and safety results. J. Cardiovasc. Electrophysiol. 30, 918–925 (2019). 30907462 10.1111/jce.13927

[46] R. F. Evonich, D. M. Nori, D. E. Haines, Efficacy of pulmonary vein isolation with a novel hot balloon ablation catheter. J. Interv. Card. Electrophysiol. 34, 29–36 (2012). 22228411 10.1007/s10840-011-9646-1

[47] G. S. Dhillon, S. Honarbakhsh, A. Di Monaco, A. E. Coling, K. Lenka, F. Pizzamiglio, R. J. Hunter, R. Horton, M. Mansour, A. Natale, V. Reddy, M. Grimaldi, P. Neuzil, C. Tondo, R. J. Schilling, Use of a multi-electrode radiofrequency balloon catheter to achieve pulmonary vein isolation in patients with paroxysmal atrial fibrillation: 12-Month outcomes of the RADIANCE study. J. Cardiovasc. Electrophysiol. 31, 1259–1269 (2020). 32250514 10.1111/jce.14476

[48] T. Maurer, M. Schlüter, K.-H. Kuck, Keeping it simple: Balloon devices for atrial fibrillation ablation therapy. JACC Clin. Electrophysiol. 6, 1577–1596 (2020). 33213820 10.1016/j.jacep.2020.08.041

[49] I. Kawamura, V. Y. Reddy, B. J. Wang, S. R. Dukkipati, H. W. Chaudhry, C. G. Santos-Gallego, J. S. Koruth, Pulsed field ablation of the porcine ventricle using a focal lattice-tip catheter. Circ. Arrhythm. Electrophysiol. 15, e011120 (2022). 36074657 10.1161/CIRCEP.122.011120 PMC9794124

