# Supplementary material for: Bioinspired balloon catheter integrated with stretchable “flounder” electrodes under high voltage for uniform pulsed field ablation
Source: Sci Adv. 2024 Dec 13;10(50):eadq5822. doi: 10.1126/sciadv.adq5822 (PMC11641019; doi:10.1126/sciadv.adq5822)
Supplement: Supplementary file 1 — Figs. S1 to S16 Tables S1 to S9 Legends for movies S1 to S4 References [file sciadv.adq5822_sm.pdf]

Science Advances
AAAS

Supplementary Materials for

Bioinspired balloon catheter integrated with stretchable "flounder" electrodes under high voltage for uniform pulsed field ablation

Xuejing Shen *et al.*

Corresponding author: Yihao Chen, chenyihao92@tsinghua.edu.cn; Xue Feng, fengxue@tsinghua.edu.cn

Sci. Adv. **10**, eadq5822 (2024)
DOI: 10.1126/sciadv.adq5822

The PDF file includes:

Figs. S1 to S16
Tables S1 to S9
Legends for movies S1 to S4
References

Other Supplementary Material for this manuscript includes the following:

Movies S1 to S4

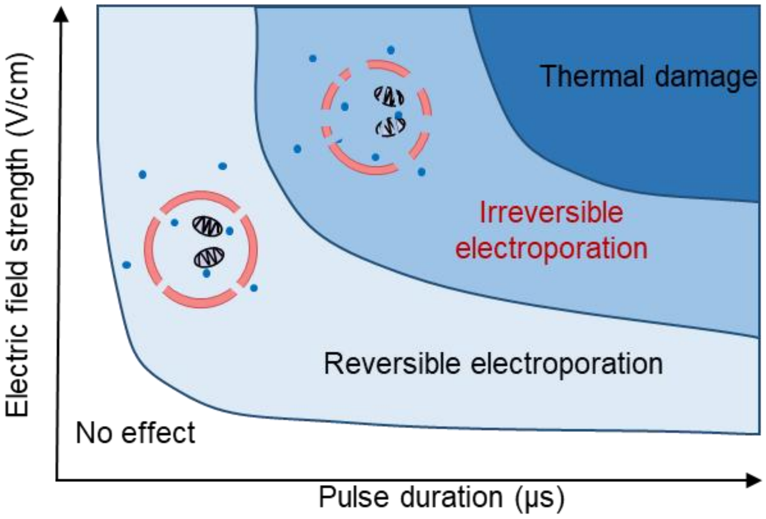

Fig. S1. The effects of pulsed electric field on the cell membrane, ranging from reversible to irreversible to thermal damage.

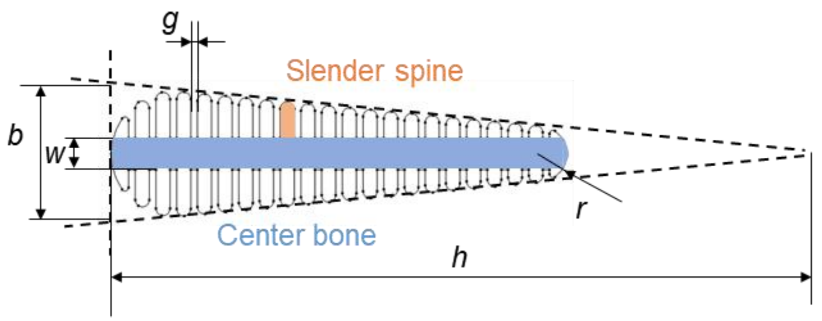

Fig. S2. Illustration of the bio-inspired "flounder" electrode design. The symbolic implication and optimized value are shown in Table S3.

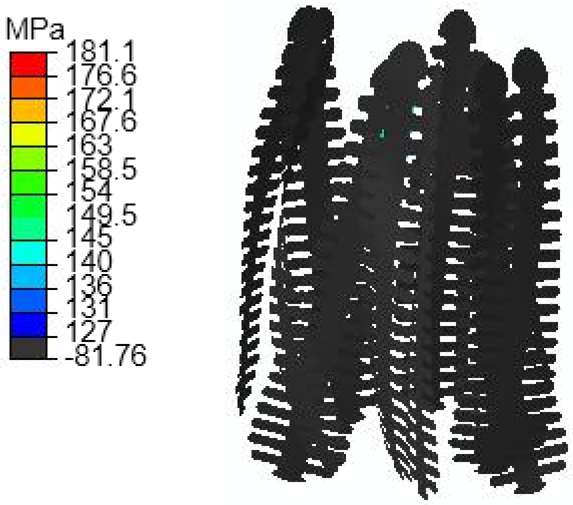

Fig. S3. Stress distribution of the electrodes with the balloon deflated 87%. Four electrodes exhibited units that exceeded the tensile strength of 127 MPa, resulting in 3, 3, 5, and 25 failed elements, respectively. The total number of units in each electrode was 3283, which means that the failed units accounted for only 0.0841% of the total.

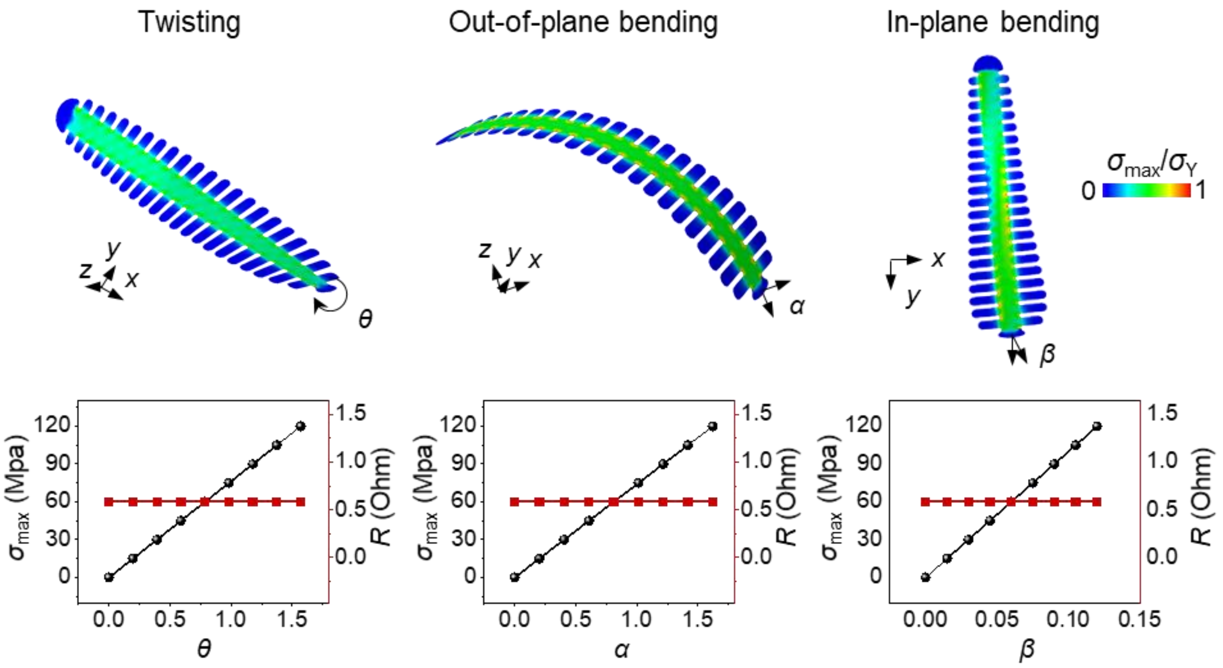

Fig. S4. Mechanical analysis and the electrical responses of the electrode unit under twisting, out-of-plane bending and in-plane bending. The maximum twisting angle, out-of-plane bending angle, and in-plane bending angle were measured as 90.0°, 92.8°, and 6.8°, respectively. The stable resistance responses of the electrode unit under different loading conditions demonstrate the robustness of the fabricated device.

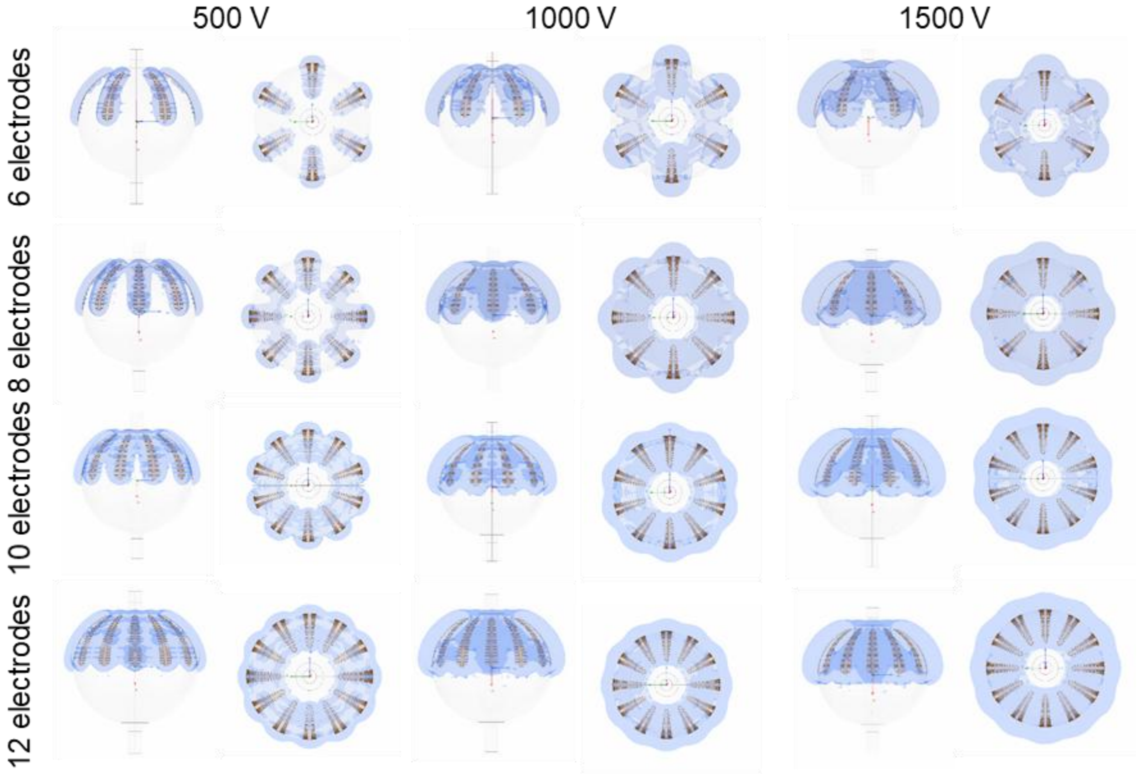

Fig S5. The top and side views of the electrostatic field. Electrostatic fields excited by balloon electrode with different number of electrodes and exciting voltages. The boundary is 400 V/cm.

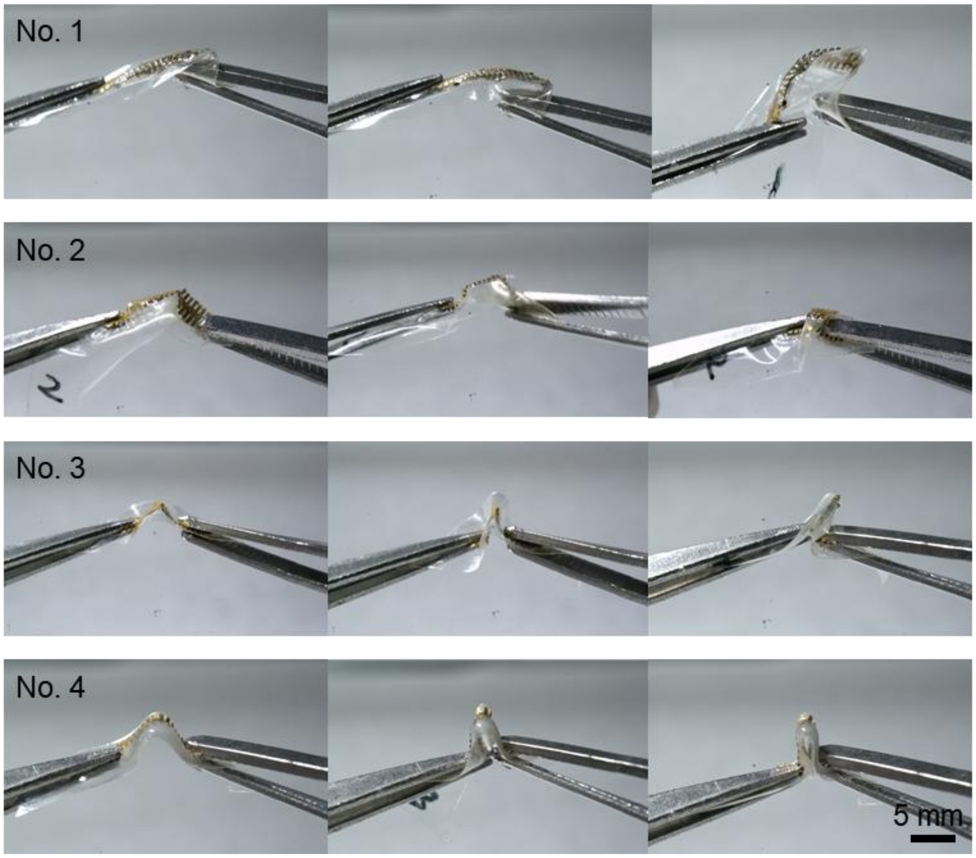

Fig. S6. Bending tests of electrodes attached to balloon slice with different adhesives.

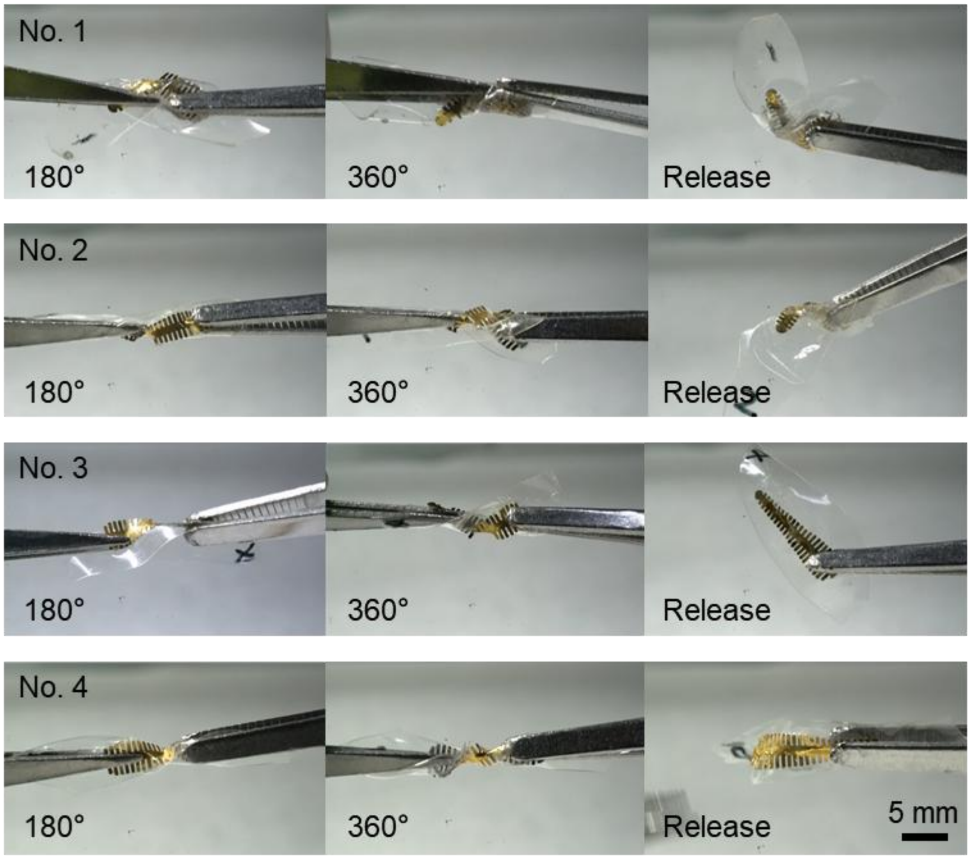

Fig. S7. Twisting tests of electrodes attached to balloon slice with different adhesives. The No.1 and No.2 adhesives failure to revert to their original states after twisting test.

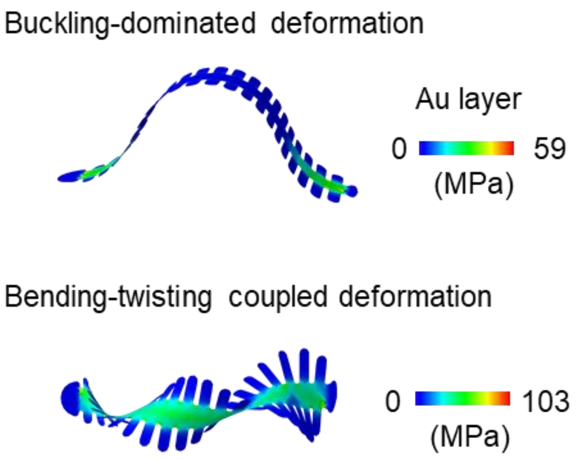

Fig. S8. FEA results of the electrode unit under the buckling-dominated deformation and bending-twisting coupled deformation.

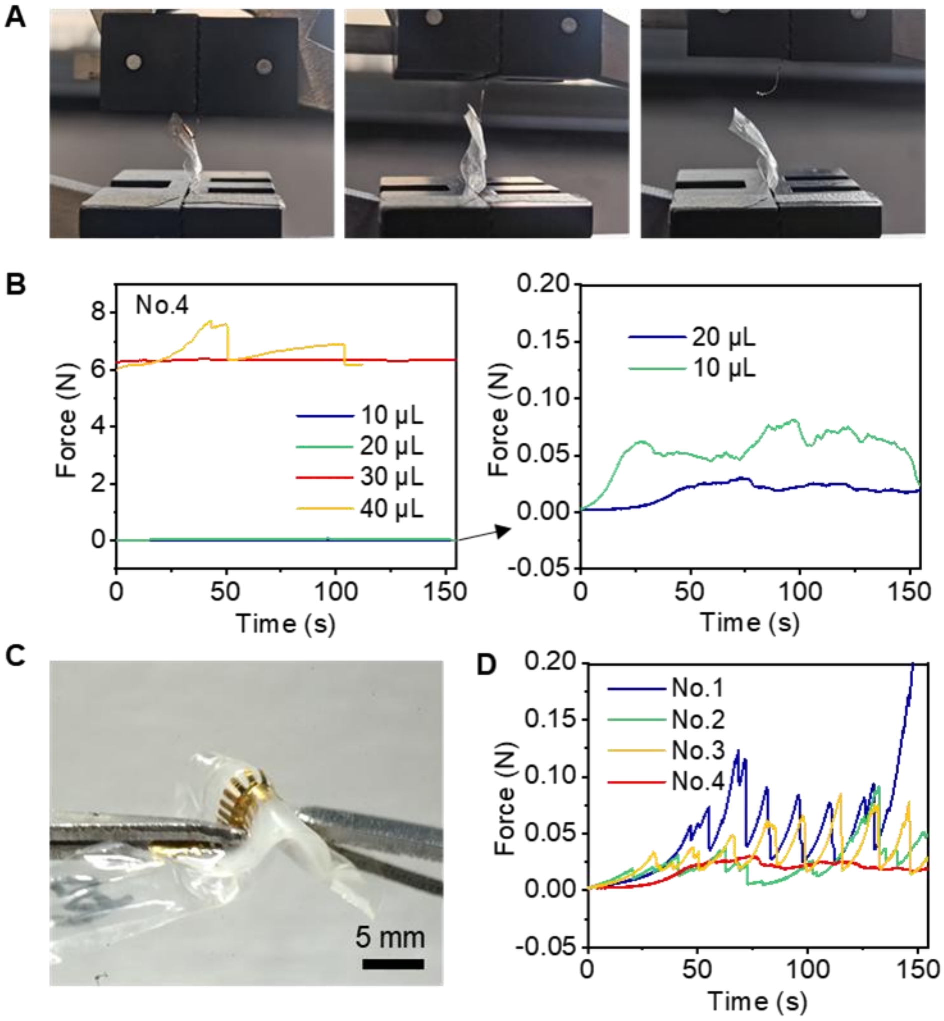

Fig. S9. The effect of adhesives on the interface of the electrode and the balloon. (A) The process of peeling. **(B)** The curves of peel strength for No.4 adhesive with amounts ranging from 10 to 40 μL. (**C**) Excessive the No.4 adhesive with no effect on deformation (the amount of 40 μL). (**D**) The curves of peel strength for different adhesives with same amount of 20 μL.

Common knowledge suggests that increasing the amount of adhesive generally enhances the bonding strength; but increased adhesive easily leads localized hardening that impairs the deformability of the balloon. Here, an increase in the amount of adhesive substantially enhanced the peel strength. For instance, when the amount of adhesive increased from 10 μL to 30 μL, the peel strength jumped from 0.05 N to 6.4 N with an increase of ~130%. Moreover, an increase in the usage of adhesive did not affect the electrode deformation (fig. S6C), where favorable adhesion and optimal flexibility of the specimen were observed. The No.4 adhesion provides great flexibility, smooth bending, and strong adhesion, fulfilling the requisite for flexible electrodes integrated the balloon catheter.

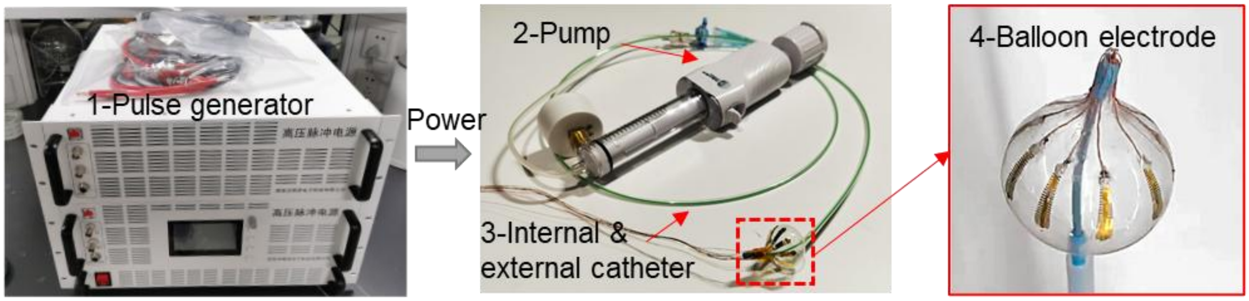

Fig. S10. The complete balloon-based PFA system. A balloon-based PFA system includes basics four parts: 1) the self-developed pulse generator, 2) a pump, 3) the multi-lumen catheter, and 4) the balloon electrode.

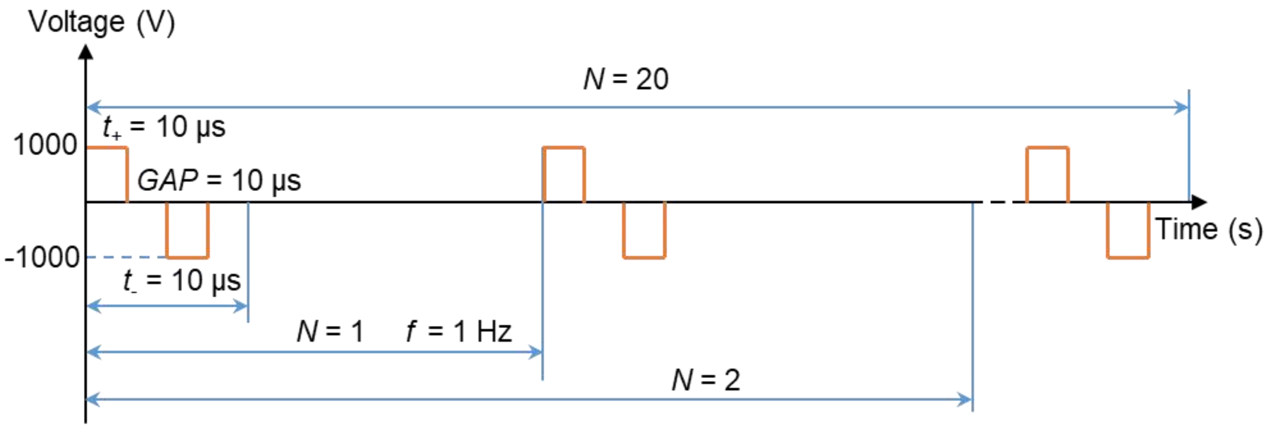

Fig. S11. The illustration of optimized pulse parameters. *t* represents the pulse width, which refers to the duration of each individual pulse in a sequence. *N* denotes the pulse number, indicating the total number of pulses delivered during the experiment or treatment. *f* corresponds to the frequency, defined as the number of pulses occurring per unit of time.

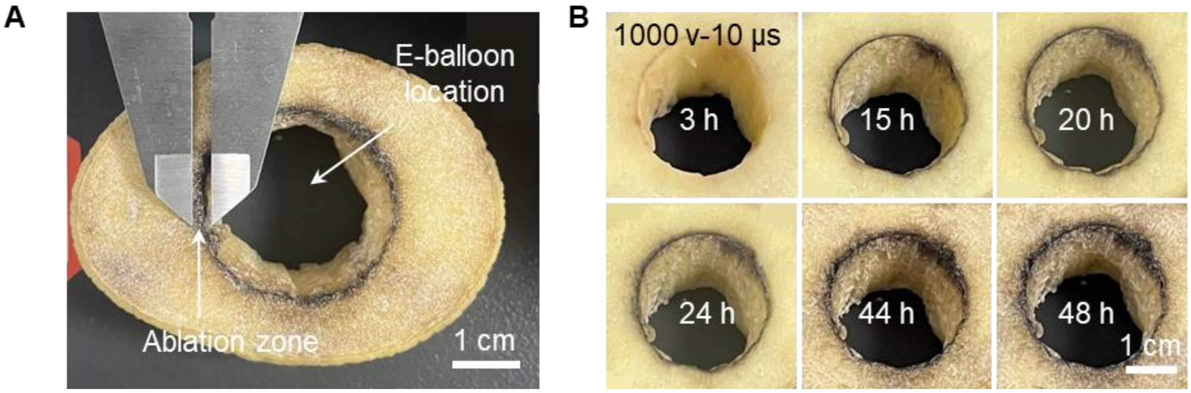

Fig. S12. Measurement and evolution of ablation depth with time in potato experiment. (A) The measurement of ablation depth in potato experiment. **(B)** The photos from a potato model under PFA with voltage of 1000 V and pulse width of 10 μs after 3 h to 48 h.

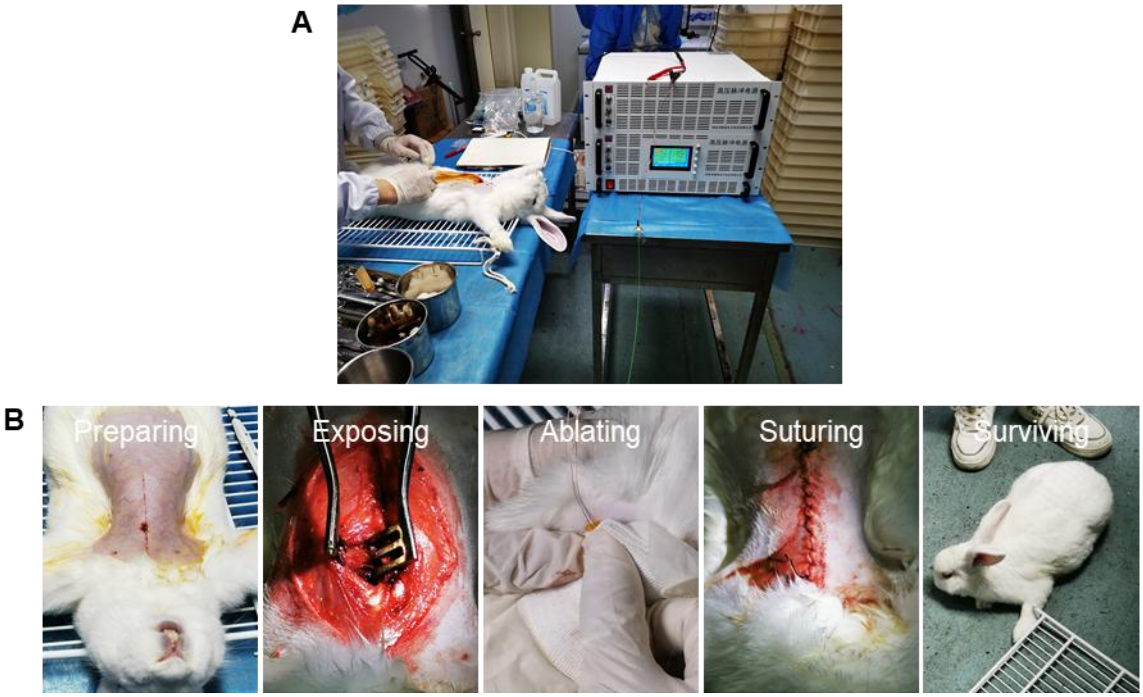

Fig. S13. Surgery scene of rabbit experiment. (**A**) The photo taken during the rabbit experiment. (**B**) The procedure of the rabbit experiment.

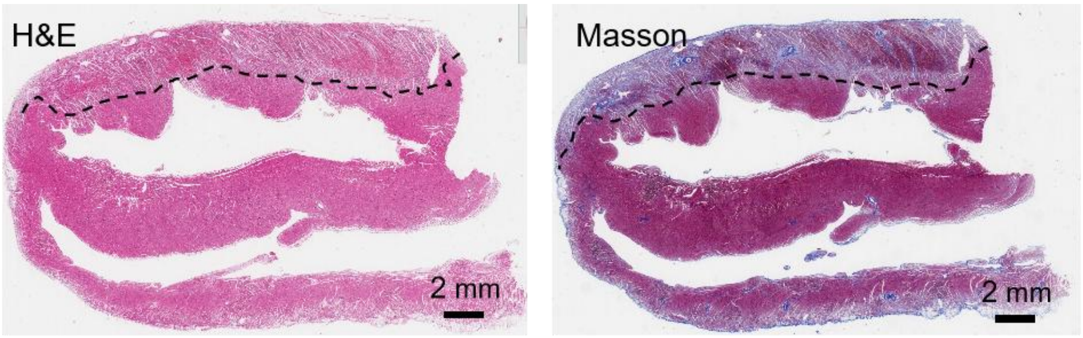

Fig. S14. Histological sections of myocardial tissues after PFA from a rabbit. The tissue underwent H&E and Masson's trichrome stains. The dashed line indicates ablation lesion boundary.

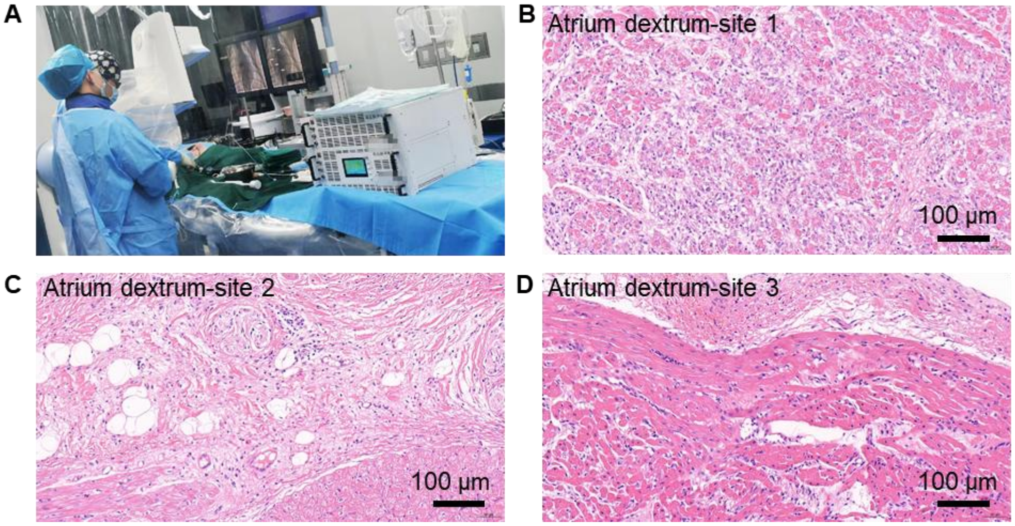

Fig. S15. The photo and histological sections from the swine experiment. (**A**) The photo taken during the swine experiment. (**C-D**) Histological sections from atrium dextrum after PFA.

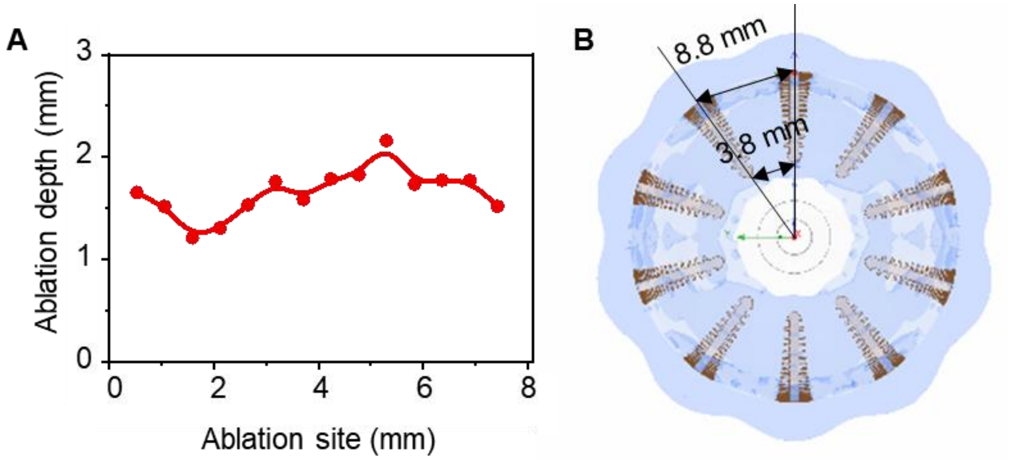

Fig. S16. Uniform ablation depths across distance of electrodes. (**A**) The ablation boundary curve from Fig. 5H in manuscript. (**B**) A balloon model with 10 skeletons showing distance between two skeletons.

Table S1. Comparison of catheter ablation techniques of atrial fibrillation.

**Ablation technique**	Cryoablation	Radiofrequency (RF)	Pulsed field ablations (PFA)
**Energy source**	Thermal energy	Thermal energy	Pulsed electric filed
**Control method**	Temperature	Power	Voltage
**Contributing factor**	Temperature (Cooling rate, Target temperature, Time at target temperature, Thawing rate)	Current ($I^2=P/R$); (I-current, P-power, R-impedance)	Charge (Voltage, pulse width, pulse number)
**Voltage**	-	~100 V	500~3000 V
**Frequency**	-	Up to 500 kHz	1~10 Hz
**Temperature**	－20℃	＞45℃	＜45℃
**Tissue selectivity**	None	None	Yes
**Ref**	[15]	[7]	[44]

Table S2. Comparison of balloon device for catheter ablation of atrial fibrillation.

**Device**	*Arctic Front / POLARx*	*HotBalloon*	*Heliostar*	*Luminize*	Bio-inspired Balloon Catheter Integrated Flounder Electrodes
**Energy source**	Cryoenergy	RF	RF	RF	PFA
**Voltage (V)**	-	*122	*39	*33	1200
**Power (W)**	-	150	15	11	0.2
**Electrode shape**	-	Coil	Patterned electrodes	Plate	Fishbone-like electrodes
**Electrode distribution**	-	Central	Circumferential	Hemispherical	Circumferential
**Electrode number**	-	1	10	18	10
**Electrode stretchability**	-	No	No	No	Yes
**Tissue selectivity**	No	No	No	No	Yes
**Ref**	[45]	[46]	[47]	[48]	Our work

* The value is calculated according to the tissue impedance of 100 ohms.

Table S3. The geometric parameters of the bio-inspired "flounder" electrode design.

Symbol	Implication	Value
b	Base of the triangle	3.5 mm
h	Height of the triangle	18.6 mm
w	Bone width	0.8 mm
g	Gap width	0.17 mm
n	Number of gaps	22
r	Fillet radius	0.72 mm

Table S4. The ablation depth (mm) in the electric field simulation.

Voltage (V)	Site	Electrodes number			
		6	8	10	12
500	30°	1.63	1.80	1.50	1.90
	60°	1.97	2.00	2.00	2.24
	90°	1.85	1.90	2.00	2.06
1000	30°	2.79	3.03	2.89	2.68
	60°	3.55	3.57	3.4	3.57
	90°	3.00	3.00	3.00	3.15
1500	30°	4.16	3.65	3.38	3.14
	60°	4.65	4.63	4.16	4.28
	90°	4.10	4.05	4.07	4.06
500	Max	1.90	1.89	2.00	2.07
	Min	0.00	0.00	0.00	1.04
1000	Max	3.27	3.22	3.3	3.29
	Min	0.00	0.85	2.16	2.69
1500	Max	4.27	4.2	4.17	4.07
	Min	0.36	2.6	3.42	3.62

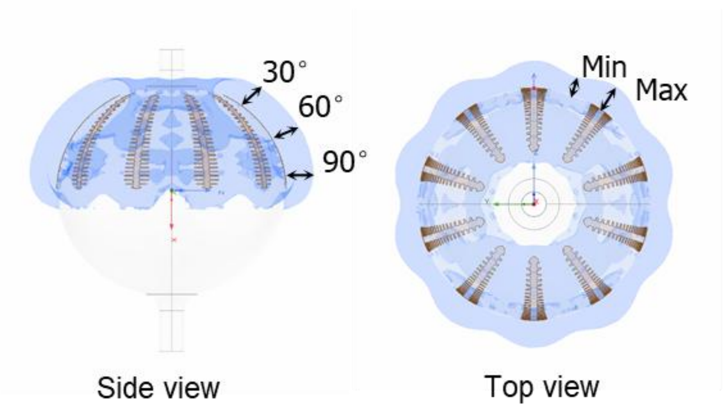

Table S5. Peel strength of different adhesives.

Adhesive	No.1	No.2	No.3	No.4	No.4	No.4	No.4
**Amount (uL)**	20	20	20	20	10	30	40
**Average (N)**	0.082	0.025	0.029	0.018	0.052	6.35	6.654
**Max (N)**	0.5407	0.1028	0.08488	0.0303	0.08157	6.388	7.73

Table S6. The ablation depths in potato experiments.

**Voltage (V)**	400	600	800	1000	1100	1200	1000	1000	1000	1000	1000	1000
**Width (μs)**	10	10	10	10	10	10	1	5	10	15	20	25
**Pulse number**	20	20	20	20	20	20	20	20	20	20	20	20
**Ablation depth (mm)**	0.52	0.56	1.54	2.21	2.64	2.67	1.00	1.74	2.64	3.42	3.73	3.80

Table S7. The ablation depths of thigh muscle in rabbit experiments.

**Voltage(V)**	600	800	800	800	1000
**Pulse number**	10	10	30	50	10
**Ablation depth (mm)**	0	0	0.95	2.77	3.1

Table S8. The ablation depth from different sections in swine experiments.

Section	PFA parameter	Ablation depth (mm）
Right upper pulmonary vein	1000 V, 10 μs, 25 numbers	1.8102
Atrium dextrum-site1	1000 V, 10 μs, 25 numbers + 1000 V, 20 μs, 20 numbers	2.1765
Atrium dextrum-site 2		0
Atrium dextrum-site 3		2.3277

Table S9. Comparison of the bio-inspired balloon catheter with other clinical PFA catheters.

Ref	Electric field direction (Diameter)	Morphology	Hardness	Voltage (V)	Shot times	Lesion depth (mm)	Lesion depth per shot (mm)	Lesion width (mm)	Lesion width per shot (mm)
Our work	Unidirectional (28 mm)	Balloon: 10 flounder electrodes	Soft	800~ 1200	1x @rabbit<br>2x @swine	3.1 @rabbit<br>2.3 @swine	3.1 @rabbit<br>1.15 @swine	> 8	> 8
Sphere 9 [49]	Non-unidirectional (9 mm)	Focal lattice	Rigid	2000	2x @swine	4.2 @swine	2.1 @swine	10.5 @swine	5.3 @swine
Globe [31]	Unidirectional (30 mm)	Basket	Rigid	1600~ 2000	1x @canine	2.51 @canine	2.51 @canine	-	-
FaraPulse [27]	Non-unidirectional (31/35 mm)	Spline: 5 splines x 4 electrodes	Rigid	800	4x @swine	0.83 @ swine	0.2 @ swine	-	-
[28]	Non-unidirectional	Spline: 4 splines x 3 electrodes	Rigid	2200	6x @swine	6.5 @ swine	1.08 @swine	22.6 @swine	3.78 @swine
PulseSelect [25]	Non-unidirectional (25 mm)	Circular: 9 electrodes	Rigid	500	5x @swine	2.25 @ swine	0.45 @ swine	12 @swine	2.4 @swine

Product web site:
Sphere 9: *https://news.medtronic.com/2022-12-05-Medtronic-completes-enrollment-in-pivotal-trial-evaluating-first-of-its-kind-pulsed-field-ablation-catheter-for-patients-with-atrial-fibrillation*;
Globe: https://kardium.com/globe-system/;
FaraPulse: https://www.bostonscientific.com/en-US/products/catheters--ablation/farapulse.html;
PulseSelect: *https://www.medtronic.com/us-en/healthcare-professionals/products/cardiac-rhythm/ablation-atrial-fibrillation/pulseselect-pulsed-field-ablation-system.html*

Movie S1. Contraction process of the e-balloon simulated by finite element analysis.

Movie S2. Inflating process of an e-balloon deployed from 14F mock catheter.

Movie S3. Fatigue test of e-balloon inflating, deflating and moving in mock catheter.

Movie S4. PFA process in thigh muscle of a rabbit.

REFERENCES AND NOTES

1. G. Y. H. Lip, H. F. Tse, D. A. Lane, Atrial fibrillation. *Lancet* **379**, 648–661 (2012).

2. A. D. Elliott, M. E. Middeldorp, I. C. Van Gelder, C. M. Albert, P. Sanders, Epidemiology and modifiable risk factors for atrial fibrillation. *Nat. Rev. Cardiol.* **20**, 404–417 (2023).

3. H. Calkins, G. Hindricks, R. Cappato, Y.-H. Kim, E. B. Saad, L. Aguinaga, J. G. Akar, V. Badhwar, J. Brugada, J. Camm, P.-S. Chen, S.-A. Chen, M. K. Chung, J. Cosedis Nielsen, A. B. Curtis, D. W. Davies, J. D. Day, A. d'Avila, N. M. S. de Groot, L. Di Biase, M. Duytschaever, J. R. Edgerton, K. A. Ellenbogen, P. T. Ellinor, S. Ernst, G. Fenelon, E. P. Gerstenfeld, D. E. Haines, M. Haissaguerre, R. H. Helm, E. Hylek, W. M. Jackman, J. Jalife, J. M. Kalman, J. Kautzner, H. Kottkamp, K. H. Kuck, K. Kumagai, R. Lee, T. Lewalter, B. D. Lindsay, L. Macle, M. Mansour, F. E. Marchlinski, G. F. Michaud, H. Nakagawa, A. Natale, S. Nattel, K. Okumura, D. Packer, E. Pokushalov, M. R. Reynolds, P. Sanders, M. Scanavacca, R. Schilling, C. Tondo, H.-M. Tsao, A. Verma, D. J. Wilber, T. Yamane, C. Blomström-Lundqvist, A. A. V. De Paola, P. M. Kistler, G. Y. H. Lip, N. S. Peters, C. F. Pisani, A. Raviele, E. B. Saad, K. Satomi, M. K. Stiles, S. Willems, 2017 HRS/EHRA/ECAS/APHRS/SOLAECE expert consensus statement on catheter and surgical ablation of atrial fibrillation. *Europace* **20**, e1-e160 (2018).

4. D. S. Chew, E. Black-Maier, Z. Loring, P. A. Noseworthy, D. L. Packer, D. V. Exner, D. B. Mark, J. P. Piccini, Diagnosis-to-ablation time and recurrence of atrial fibrillation following catheter ablation. *Circ. Arrhythm. Electrophysiol.* **13**, e008128 (2020).

5. J. L. Pallisgaard, G. H. Gislason, J. Hansen, A. Johannessen, C. Torp-Pedersen, P. V. Rasmussen, M. L. Hansen, Temporal trends in atrial fibrillation recurrence rates after ablation between 2005 and 2014: A nationwide Danish cohort study. *Eur. Heart J.* **39**, 442–449 (2018).

6. D. Spragg, Collateral damage during ablation of atrial fibrillation—Lessons learnt in the past decade. *J. Atr. Fibrillation* **4**, 478 (2012).

7. M. El Baba, D. Sabayon, M. Refaat, Radiofrequency catheter ablation: How to manage and prevent collateral damage? *J. Innov. Card. Rhythm Manag.* **11**, 4234–4240 (2020).

8. H.-S. Mun, B. Joung, J. Shim, H. J. Hwang, J. Y. Kim, M.-H. Lee, H.-N. Pak, Does additional linear ablation after circumferential pulmonary vein isolation improve clinical outcome in patients with paroxysmal atrial fibrillation? Prospective randomised study. *Heart* **98**, 480–484 (2012).

9. A. Verma, C.-y. Jiang, T. R. Betts, J. Chen, I. Deisenhofer, R. Mantovan, L. Macle, C. A. Morillo, W. Haverkamp, R. Weerasooriya, J.-P. Albenque, S. Nardi, E. Menardi, P. Novak, P. Sanders, STAR AF II Investigators, Approaches to catheter ablation for persistent atrial fibrillation. *N. Engl. J. Med.* **372**, 1812–1822 (2015).

10. M. R. Williams, J. R. Stewart, S. F. Bolling, S. Freeman, J. T. Anderson, M. Argenziano, C. R. Smith, M. C. Oz, Surgical treatment of atrial fibrillation using radiofrequency energy. *Ann. Thorac. Surg.* **71**, 1939–1944 (2001).

11. A. Njoku, M. Kannabhiran, R. Arora, P. Reddy, R. Gopinathannair, D. Lakkireddy, P. Dominic, Left atrial volume predicts atrial fibrillation recurrence after radiofrequency ablation: A meta-analysis. *Europace* **20**, 33–42 (2018).

12. K.-H. Kuck, J. Brugada, A. Fürnkranz, A. Metzner, F. Ouyang, K. R. J. Chun, A. Elvan, T. Arentz, K. Bestehorn, S. J. Pocock, J.-P. Albenque, C. Tondo, for the FIRE AND ICE Investigators, Cryoballoon or radiofrequency ablation for paroxysmal atrial fibrillation. *N. Engl. J. Med.* **374**, 2235–2245 (2016).

13. J. G. Andrade, P. Khairy, P. G. Guerra, M. W. Deyell, L. Rivard, L. Macle, B. Thibault, M. Talajic, D. Roy, M. Dubuc, Efficacy and safety of cryoballoon ablation for atrial fibrillation: A systematic review of published studies. *Heart Rhythm* **8**, 1444–1451 (2011).

14. J. G. Andrade, Cryoballoon ablation for pulmonary vein isolation. *J. Cardiovasc. Electrophysiol.* **31**, 2128–2135 (2020).

15. J. P. Erinjeri, T. W. I. Clark, Cryoablation: Mechanism of action and devices. *J. Vasc. Interv. Radiol.* **21**, S187–S191 (2010).

16. A. Verma, S. J. Asivatham, T. Deneke, Q. Castellvi, R. E. Neal II, Primer on pulsed electrical field ablation: Understanding the benefits and limitations. *Circ. Arrhythm. Electrophysiol.* **14**, e010086 (2021).

17. V. Y. Reddy, P. Neuzil, J. S. Koruth, J. Petru, M. Funosako, H. Cochet, L. Sediva, M. Chovanec, S. R. Dukkipati, P. Jais, Pulsed field ablation for pulmonary vein isolation in atrial fibrillation. *J. Am. Coll. Cardiol.* **74**, 315–326 (2019).

18. C. Gianni, Q. Chen, D. Della Rocca, U. Canpolat, H. Ayhan, B. MacDonald, S. Mohanty, C. Trivedi, A. Natale, A. Al-Ahmad, Radiofrequency balloon devices for atrial fibrillation ablation. *Card. Electrophysiol. Clin.* **11**, 487–493 (2019).

19. D.-H. Kim, N. Lu, R. Ghaffari, Y.-S. Kim, S. P. Lee, L. Xu, J. Wu, R.-H. Kim, J. Song, Z. Liu, J. Viventi, B. de Graff, B. Elolampi, M. Mansour, M. J. Slepian, S. Hwang, J. D. Moss, S.-M. Won, Y. Huang, B. Litt, J. A. Rogers, Materials for multifunctional balloon catheters with capabilities in cardiac electrophysiological mapping and ablation therapy. *Nat. Mater.* **10**, 316–323 (2011).

20. M. Han, L. Chen, K. Aras, C. Liang, X. Chen, H. Zhao, K. Li, N. R. Faye, B. Sun, J. H. Kim, W. Bai, Q. Yang, Y. Ma, W. Lu, E. Song, J. M. Baek, Y. Lee, C. Liu, J. B. Model, G. Yang, R. Ghaffari, Y. Huang, I. R. Efimov, J. A. Rogers, Catheter-integrated soft multilayer electronic arrays for multiplexed sensing and actuation during cardiac surgery. *Nat. Biomed. Eng.* **4**, 997–1009 (2020).

21. A. Deshmukh, N. J. Patel, S. Pant, N. Shah, A. Chothani, K. Mehta, P. Grover, V. Singh, S. Vallurupalli, G. T. Savani, A. Badheka, T. Tuliani, K. Dabhadkar, G. Dibu, Y. M. Reddy, A. Sewani, M. Kowalski, R. Mitrani, H. Paydak, J. F. Viles-Gonzalez, In-hospital complications associated with catheter ablation of atrial fibrillation in the United States between 2000 and 2010. *Circulation* **128**, 2104–2112 (2013).

22. M. Mansour, D. Lakkireddy, D. Packer, J. D. Day, S. Mahapatra, K. Brunner, V. Reddy, A. Natale, Safety of catheter ablation of atrial fibrillation using fiber optic–based contact force sensing. *Heart Rhythm* **14**, 1631–1636 (2017).

23. A. Verma, L. Boersma, D. E. Haines, A. Natale, F. E. Marchlinski, P. Sanders, H. Calkins, D. L. Packer, J. Hummel, B. Onal, S. Rosen, K.-H. Kuck, G. Hindricks, B. Wilsmore, First-in-human experience and acute procedural outcomes using a novel pulsed field ablation system: The PULSED AF Pilot Trial. *Circ. Arrhythm. Electrophysiol.* **15**, e010168 (2022).

24. F. H. M. Wittkampf, R. van Es, K. Neven, Electroporation and its relevance for cardiac catheter ablation. *JACC Clin. Electrophysiol.* **4**, 977–986 (2018).

25. M. T. Stewart, D. E. Haines, A. Verma, N. Kirchhof, N. Barka, E. Grassl, B. Howard, Intracardiac pulsed field ablation: Proof of feasibility in a chronic porcine model. *Heart Rhythm* **16**, 754–764 (2019).

26. V. Y. Reddy, J. Koruth, P. Jais, J. Petru, F. Timko, I. Skalsky, R. Hebeler, L. Labrousse, L. Barandon, S. Kralovec, M. Funosako, B. B. Mannuva, L. Sediva, P. Neuzil, Ablation of atrial fibrillation with pulsed electric fields: An ultra-rapid, tissue-selective modality for cardiac ablation. *JACC Clin. Electrophysiol.* **4**, 987–995 (2018).

27. J. Koruth, K. Kuroki, J. Iwasawa, Y. Enomoto, R. Viswanathan, R. Brose, E. D. Buck, M. Speltz, S. R. Dukkipati, V. Y. Reddy, Preclinical evaluation of pulsed field ablation: Electrophysiological and histological assessment of thoracic vein isolation. *Circ. Arrhythm. Electrophysiol.* **12**, e007781 (2019).

28. J. S. Koruth, K. Kuroki, J. Iwasawa, R. Viswanathan, R. Brose, E. D. Buck, E. Donskoy, S. R. Dukkipati, V. Y. Reddy, Endocardial ventricular pulsed field ablation: A proof-of-concept preclinical evaluation. *Europace* **22**, 434–439 (2020).

29. V. Y. Reddy, E. Anter, G. Rackauskas, P. Peichl, J. S. Koruth, J. Petru, M. Funasako, K. Minami, A. Natale, P. Jais, H. Nakagawa, G. Marinskis, A. Aidietis, J. Kautzner, P. Neuzil, Lattice-tip focal ablation catheter that toggles between radiofrequency and pulsed field energy to treat atrial fibrillation. *Circ. Arrhythm. Electrophysiol.* **13**, e008718 (2020).

30. H. Kottkamp, F. Moser, A. Rieger, D. Schreiber, C. Pönisch, M. Trofin, Global multielectrode contact mapping plus ablation with a single catheter: Preclinical and preliminary experience in humans with atrial fibrillation. *J. Cardiovasc. Electrophysiol.* **28**, 1247–1256 (2017).

31. J. Koruth, A. Verma, I. Kawamura, D. Reinders, J. G. Andrade, M. W. Deyell, N. Mehta, V. Y. Reddy, PV isolation using a spherical array PFA catheter: Preclinical assessment and comparison to radiofrequency ablation. *JACC Clin. Electrophysiol.* **9**, 652–666 (2023).

32. L. Lu, S. Leanza, R. R. Zhao, Origami with rotational symmetry: A review on their mechanics and design. *Appl. Mech. Rev.* **75**, 050801 (2023).

33. S. P. M. Bane, J. L. Ziegler, J. E. Shepherd, Investigation of the effect of electrode geometry on spark ignition. *Combust. Flame* **162**, 462–469 (2015).

34. Y. Su, Z. Liu, S. Wang, R. Ghaffari, D.-H. Kim, K.-C. Hwang, J. A. Rogers, Y. Huang, Mechanics of stretchable electronics on balloon catheter under extreme deformation. *Int. J. Solids Struct.* **51**, 1555–1561 (2014).

35. C. R. Black, P. B. Berendzen, Shared ecological traits influence shape of the skeleton in flatfishes (Pleuronectiformes). *PeerJ* **8**, e8919 (2020).

36. G. E. Dieter, D. Bacon, *Mechanical Metallurgy* (McGraw-hill New York, 1976), vol. **3**.

37. I. Kaminska, M. Kotulska, A. Stecka, J. Saczko, M. Drag-Zalesinska, T. Wysocka, A. Choromanska, N. Skolucka, R. Nowicki, J. Marczak, J. Kulbacka, Electroporation-induced changes in normal immature rat myoblasts (H9C2). *Gen. Physiol. Biophys.* **31**, 19–25 (2012).

38. P. J. Kelly, R. D. Arnell, Magnetron sputtering: A review of recent developments and applications. *Vacuum* **56**, 159–172 (2000).

39. X. Chen, W. Jian, Z. Wang, J. Ai, Y. Kang, P. Sun, Z. Wang, Y. Ma, H. Wang, Y. Chen, X. Feng, Wrap-like transfer printing for three-dimensional curvy electronics. *Sci. Adv.* **9**, eadi0357 (2023).

40. M. Hjouj, B. Rubinsky, Magnetic resonance imaging characteristics of nonthermal irreversible electroporation in vegetable tissue. *J. Membr. Biol.* **236**, 137–146 (2010).

41. W. Lee, S. Kobayashi, M. Nagase, Y. Jimbo, I. Saito, Y. Inoue, T. Yambe, M. Sekino, G. G. Malliaras, T. Yokota, M. Tanaka, T. Someya, Nonthrombogenic, stretchable, active multielectrode array for electroanatomical mapping. *Sci. Adv.* **4**, eaau2426 (2018).

42. H. Hu, Y. Ma, X. Gao, D. Song, M. Li, H. Huang, X. Qian, R. Wu, K. Shi, H. Ding, M. Lin, X. Chen, W. Zhao, B. Qi, S. Zhou, R. Chen, Y. Gu, Y. Chen, Y. Lei, C. Wang, C. Wang, Y. Tong, H. Cui, A. Abdal, Y. Zhu, X. Tian, Z. Chen, C. Lu, X. Yang, J. Mu, Z. Lou, M. Eghtedari, Q. Zhou, A. Oberai, S. Xu, Stretchable ultrasonic arrays for the three-dimensional mapping of the modulus of deep tissue. *Nat. Biomed. Eng.* **7**, 1321–1334 (2023).

43. S. Chen, J. Qi, S. Fan, Z. Qiao, J. C. Yeo, C. T. Lim, Flexible wearable sensors for cardiovascular health monitoring. *Adv. Healthc. Mater.* **10**, e2100116 (2021).

44. R. V. Davalos, I. L. M. Mir, B. Rubinsky, Tissue ablation with irreversible electroporation. *Ann. Biomed. Eng.* **33**, 223–231 (2005).

45. F. Straube, U. Dorwarth, J. Pongratz, B. Bruck, M. Wankerl, S. Hartl, E. Hoffmann, The fourth cryoballoon generation with a shorter tip to facilitate real-time pulmonary vein potential recording: Feasibility and safety results. *J. Cardiovasc. Electrophysiol.* **30**, 918–925 (2019).

46. R. F. Evonich, D. M. Nori, D. E. Haines, Efficacy of pulmonary vein isolation with a novel hot balloon ablation catheter. *J. Interv. Card. Electrophysiol.* **34**, 29–36 (2012).

47. G. S. Dhillon, S. Honarbakhsh, A. Di Monaco, A. E. Coling, K. Lenka, F. Pizzamiglio, R. J. Hunter, R. Horton, M. Mansour, A. Natale, V. Reddy, M. Grimaldi, P. Neuzil, C. Tondo, R. J. Schilling, Use of a multi-electrode radiofrequency balloon catheter to achieve pulmonary vein isolation in patients with paroxysmal atrial fibrillation: 12-Month outcomes of the RADIANCE study. *J. Cardiovasc. Electrophysiol.* **31**, 1259–1269 (2020).

48. T. Maurer, M. Schlüter, K.-H. Kuck, Keeping it simple: Balloon devices for atrial fibrillation ablation therapy. *JACC Clin. Electrophysiol.* **6**, 1577–1596 (2020).

49. I. Kawamura, V. Y. Reddy, B. J. Wang, S. R. Dukkipati, H. W. Chaudhry, C. G. Santos-Gallego, J. S. Koruth, Pulsed field ablation of the porcine ventricle using a focal lattice-tip catheter. *Circ. Arrhythm. Electrophysiol.* **15**, e011120 (2022).
